# A role for the *Stentor* syntaxin protein in post-wound cell survival

**DOI:** 10.1091/mbc.E25-11-0527

**Published:** 2026-07-15

**Authors:** Ambika V. Nadkarni, Ulises Diaz, Ramon Rodriguez, Kevin S. Zhang, Sindy K.Y. Tang, Wallace F. Marshall

**Affiliations:** ^a^Department of Mechanical Engineering, Stanford University, Stanford, CA 94305.; ^b^Department of Biochemistry and Biophysics, University of California San Francisco, San Francisco, CA 94158.; UT Southwestern Medical Center

## Abstract

Wound repair is an essential biological process that occurs both in tissues and single cells. In free-living, single-celled ciliates such as *Stentor coeruleus*, rapid repair from wounds is necessary to heal breaches to the plasma membrane, where any delays represent the difference between life and death. To discover novel molecular pathways that are important for healing in *Stentor*, we carried out a targeted RNA interference-based perturbation genetic screen combined with microsurgical wounding using a microfluidic guillotine to introduce reproducible bisection wounds. We identified a *Stentor* syntaxin gene that was necessary for cell survival, particularly post-wounding, with only ∼37% of syntaxin-deficient cells surviving compared with ∼98% of control cells. Syntaxin-deficient cells were more susceptible to hypoosmotic shock and became increasingly vacuolated in the hours post-wounding, eventually leading to cell death. Wounding of the cells in 75 mM sorbitol during and after bisection partially restored post-wound survival in knockdown cells. These results support the interpretation that syntaxin-deficient cells lack essential membrane fusion machinery, which manifests in vacuolar defects, and are deficient in maintaining osmotic homeostasis necessary for their survival post-wounding. This study provides a template for the discovery of new wound healing biology in emerging model systems.

## Abstract (executive summary)

- *Stentor* is a single-celled ciliate with prodigious healing ability, yet the molecular mechanisms by which the cell rapidly repairs wounds are not fully characterized.
- Through a targeted microfluidics genetic screen, we identified a novel syntaxin-like protein that is essential for *Stentor* survival post-wounding.
- Syntaxin-deficient cells were more susceptible to osmotic shock. Wounding cells in the presence of 75 mM sorbitol reduced the degree of hypoosmotic stress associated with wounding and enabled more cells to survive. Our discovery highlights the rebalancing of intracellular osmolarity as an important step in wound repair in freshwater-dwelling organisms.

## INTRODUCTION

Wound healing is a universal biological response commonly examined at the tissue level. However, wound repair also occurs in single cells, particularly those that sustain damage during normal function, such as muscle cells and neurons (Abreu-Blanco *et al.*, 2011; Tang and Marshall, 2017; Ma *et al*., 2021). In the single-cell context, the membrane and underlying structures represent an outer protective barrier to injury that must be restored after wounding. A rapid response to cellular wounding is especially important in free-living, single-celled organisms for which any delays represent the difference between life and death. The giant ciliate *Stentor coeruleus* is a cone-shaped cell that attaches to substrates with a holdfast at one end, and generates a feeding flow using a ring of cilia at the other end. Stentor cells can swim using cilia on their body wall, and can change their shape by contraction and extension using centrin- and microtubule-based cytoskeletons (refer to Marshall, 2021 for review). *S. coeruleus* is well known for its ability to repair and regenerate from drastic wounds, including from a fragment as small as 1/27th of the original cell volume (Lillie, 1896). *S. coeruleus* also heals bisection wounds at ∼ 8–80 µm^2^/s, representing one of the fastest healing rates in investigated biological systems (Zhang *et al.*, 2021). This extreme ability to repair wounds in *Stentor* thus provides a powerful avenue for the discovery of new healing mechanisms.

In eukaryotic systems, wounding repair is initiated by the influx of calcium ions, which trigger molecular pathways that promote membrane resealing. Membrane dynamics occur in conjunction with the remodeling of the underlying cytoskeletal network (Abreu-Blanco *et al.*, 2011; Sonnemann and Bement, 2011). In systems such as *Xenopus* oocytes and *Drosophila* syncytial embryos, a circumferential actomyosin ring enables wound closure through a purse-string mechanism (Bement *et al.*, 1999; Abreu-Blanco *et al.*, 2011). SNARE-dependent exocytic machinery is also necessary for wound healing, as demonstrated in systems such as starfish oocytes, sea urchin eggs, and fibroblasts (Bi *et al.*, 1995; Miyake and McNeil, 1995). Application of botulinum neurotoxins to fertilized sea urchin eggs and 3T3 fibroblasts inhibited vesicle fusion and interfered with wound healing (Steinhardt *et al.*, 1994; Bi *et al.*, 1995). However, it is not known whether these mechanisms operate to close wounds in *Stentor*.

The study of wound healing and regeneration in *Stentor* has historically been carried out via microsurgery and grafting techniques. Direct imaging of membrane and cytoskeletal components in *Stentor* remains difficult because methods for transgene expression have not yet been implemented, and *Stentor's* robust efflux system sequesters small-molecule dyes in intracellular vesicles.

To address these challenges, here we carried out an RNA-interference-based genetic screen combined with a microfluidic guillotine capable of introducing reproducible bisection wounds in a high-throughput manner to identify molecular pathways important for wound healing in *Stentor*. Using this approach, we identified a *Stentor* syntaxin that was necessary for cell survival post-wounding.

Syntaxins are components of the SNARE complex (Rothman, 1994) that enable vesicle fusion (Bennett *et al.*, 1992). Syntaxins, a family of transmembrane proteins, contain a characteristic ∼60-residue, coiled-coil domain known as the SNARE domain (Teng *et al.*, 2001). Syntaxins are present in all eukaryotes and mediate vesicle fusion in a variety of cellular compartments such as the plasma membrane, ER, Golgi, and endosomes (Teng *et al.*, 2001). Different syntaxins localize to various cellular membranes and confer specificity of vesicle fusion. Syntaxins are therefore critical for a variety of cellular processes such as neurotransmitter release, insulin release, and receptor trafficking (Bennett *et al.*, 1992; Manickam *et al.*, 2011; Chen *et al.*, 2014; Kang *et al.*, 2022). Due to their importance in exocytosis, syntaxins have also been implicated in wound healing in systems such as mouse skeletal muscle cells (syntaxin 4), the *C. elegans* epidermis (syntaxin 2), and squid giant axons (Detrait *et al.*, 2000; Meng *et al.*, 2020; Chen and Michele, 2025).

Syntaxin proteins have been studied in other ciliates within the context of secretion and exocytosis. They have been shown to be important in the biogenesis of secretory organelles in *Tetrahymena* and the exocytosis of Ɣ-amino butyric acid in *Paramecium* (Dacks and Doolittle, 2004; Kissmehl *et al.*, 2007; Ramoino *et al.*, 2010; Kaur *et al.*, 2017). In Paramecium, syntaxins have also been known to localize to a specialized organelle known as the contractile vacuole (CV; Kissmehl et al., 2007; Schönemann *et al.,* 2013). The CV is a dynamic osmoregulatory organelle that collects water from the cytoplasm through a network of vesicles and tubules, and periodically expels it into the environment through a transient fusion event with the plasma membrane. This phenomenon is particularly important for freshwater ciliates that experience osmotic influx of water from their hypoosmotic environments (Allen 2000; More *et al.*, 2024).

A role for syntaxin proteins in *Stentor* has not yet been described. We therefore carried out a domain and phylogenetic analysis of the syntaxin protein. We further identified the role of syntaxin in post-wound survival and adaptation to osmotic challenges in *Stentor*. Our results may indicate restoration of osmotic homeostasis, an important aspect of cellular wound recovery.

## RESULTS AND DISCUSSION

### An RNA interference-based survival screen for wound healing

**FIGURE 1:**
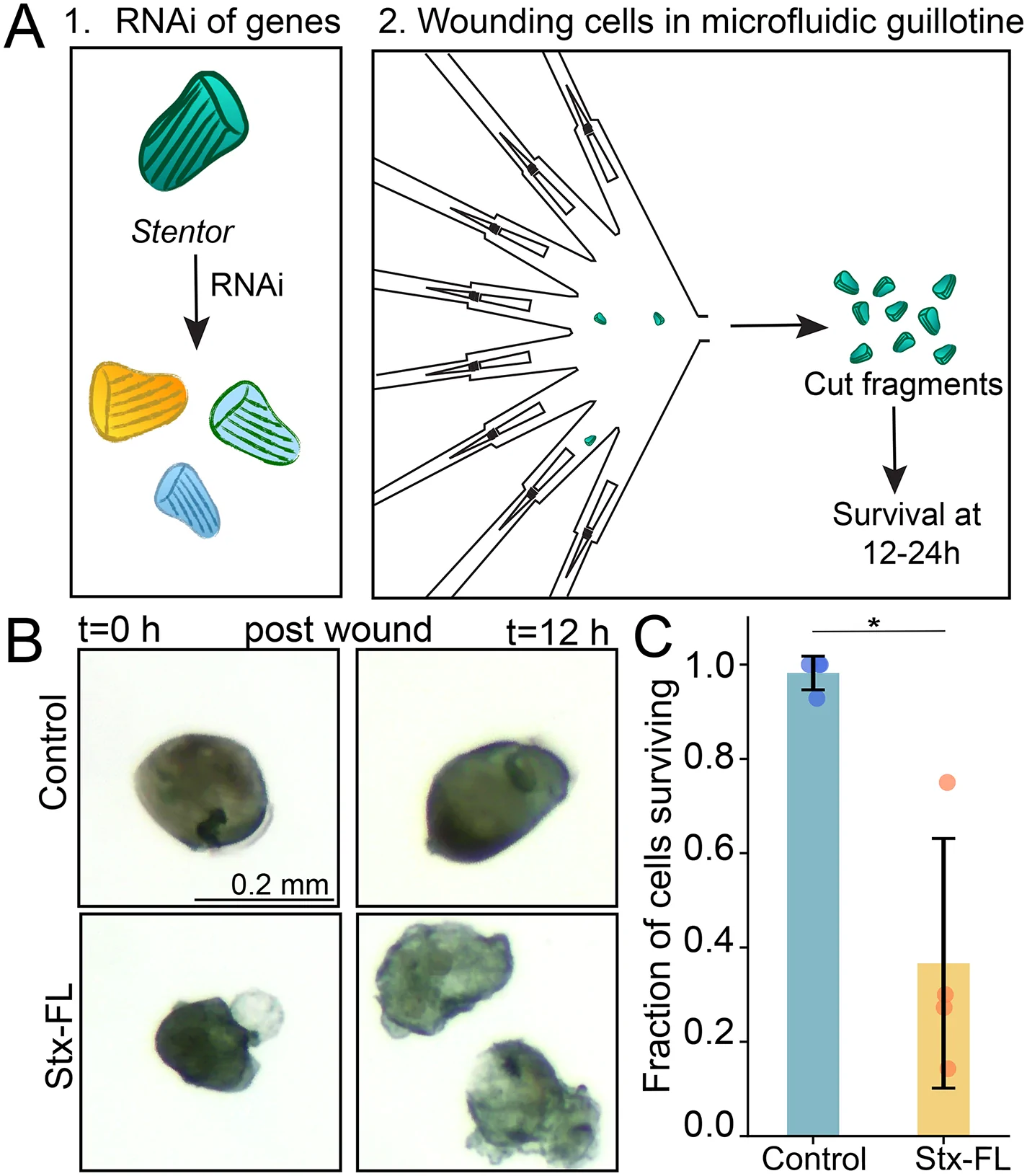
Microfluidic RNAi screen identified syntaxin as necessary for post wound cell-survival. (A) Schematic of RNAi screen.1. Left panel - Candidate genes in *Stentor coeruleus* were knocked down by feeding-based RNA interference. 2. Right panel - Survival of knocked-down cells was assayed by high-throughput bisection through a microfluidic guillotine at 12–24 h post-bisection. (B) Images of control cells knocked down for the gene Lf4 that did not impact cell survival post-bisection at 0 h (left panels) or 12 h (right panels) after wounding. Bottom panels show the identical time point for cells knocked down for the gene Stecoe_25434 (Stx-FL RNAi). (C) Quantitation of cell survival at 12 h after wounding showed the fraction of cells surviving at 12 h was 0.982 and 0.366 for control and RNAi cells, respectively (S.D. 0.036 and 0.265, *p* = 0.0265 by the non-parametric, two-sided Mann-Whitney U-test). Points on the graph indicate the survival metrics for individual experiments. *n* = 4 independent experiments, avg. 10 cell fragments per experiment.

The free-living ciliate *Stentor coeruleus* is successful at healing extreme wounds, but the molecular pathways by which this occurs are not known (Slabodnick *et al.*, 2013). We devised an RNA interference-based survival screen for wound-healing (Figure 1A). Candidate genes were selected from studies of wound healing in other biological systems, or from previous transcriptomics studies of *Stentor* (Supplemental Table S1). Gene pathways were perturbed using RNA interference (RNAi) by feeding bacteria expressing dsRNA constructs (Slabodnick *et al.*, 2014), and cells were wounded by bisection through a microfluidic guillotine as previously described (Blauch *et al.*, 2017; Koppaka *et al.*, 2021; Zhang *et al.*, 2021). Cell survival was visually determined by observing cell and ciliary movement at *t* = 0 and *t* = 12 h post-wounding. Since our goal was to perform a pilot screen to quickly prioritize targets for further study, we used a simple binary criterion for viability based on our previous work (Blauch *et al.*, 2017; Zhang *et al.*, 2021), in which we considered any movement of the oral or body cilia to signify a “live” cell, and death was therefore scored as a loss of all ciliary motility. Ciliary beating produces a characteristic helical swimming motion of cells; therefore, live and dead cells were easily distinguished. Live cell fragments also tended to adhere to the bottom of imaging wells via their “tails” or holdfasts.

*S. coeruleus* was insensitive to the perturbation of several candidate genes that have been shown to be important in other systems. Survival was unaffected by perturbation of homologs of actin (Stecoe_7446) and Cdc42 (Stecoe_21574), implicated in actin-based purse-string closure in many systems including *Xenopus* oocytes (Supplemental Figure S1; Benink and Bement, 2005).

Cells were also insensitive to perturbations of candidate homologs involved in membrane fusion and vesicle patching in other systems such as the C2 domain Ferlin protein (Stecoe_31310) and annexin (Stecoe_18601; Bansal *et al.*, 2003; McNeil *et al.*, 2006). We also tested the role of three “bisection-specific” genes (CEP19, carbonic anhydrase, and Major Facilitator Superfamily protein MFS1) in our survival assay. These genes were previously shown to be transcriptionally upregulated in both half-cells when cells were bisected, but not when the cells were subjected to non-wounding procedures such as sucrose-shock, which typically causes selective shedding of the mouth or oral apparatus (Sood *et al.*, 2022).

We did not detect any effect on post-wounding survival in cells in which actin, Cdc42, ferlin, annexin, or the bisection-specific genes CEP19 or carbonic anhydrase were knocked down (Supplemental Figure S1A). We also did not observe any obvious differences in cell size or morphology in these cells (Supplemental Figure S1B). Given the possibility of incomplete knockdown for some genes by RNAi, our results do not indicate the non-essentiality of these genes, but simply that the RNAi experiments performed here failed to produce notable survival defects post-bisection. Among these genes, knockdown of Stecoe_168 encoding Major Facilitator Superfamily protein 1 (MFS1) caused post-wounding survival defects (Supplemental Figure S1A). Major Facilitator Superfamily proteins are transmembrane transporters that mediate the transport of a variety of substrates (Pao *et al.*, 1998), but inferring substrate specificity of MFS1 from the protein sequence is beyond the scope of the current study. As a result, we have not pursued the basis of the potential decreased survival in MFS1 RNAi cells.

We observed the most consistent and statistically significant survival defect when we inhibited a syntaxin-like protein, gene Stecoe_24534 (hereafter referred to as Stx; Figure 1, B and C). Gene Lf4 was employed as a control that did not impact cell survival, as reported previously (Slabodnick *et al.*, 2014). Twelve hours post-wounding (i.e., a bisection cut; Supplemental Figure S2), only about 37% of syntaxin (full-length)-deficient (Stx-FL RNAi) cells survived, compared with about 98% of control cells surviving (*p* = 0.0265, Mann Whitney U-test; *n* = 4, where n represents the number of independent experiments, refer to *Materials and Methods* section; Figure 1C). Despite the limited replicates, the effect was statistically significant. Stx-FL RNAi cells appeared to lose membrane integrity with signs of rupture and disintegration (Figure 1B, bottom right panel), which further enabled us to distinguish “live” versus “dead” cells. We did not observe this phenomenon with any other candidate genes in our screen.

### Investigating the domain structure and phylogeny of *Stentor* syntaxin

**FIGURE 2:**
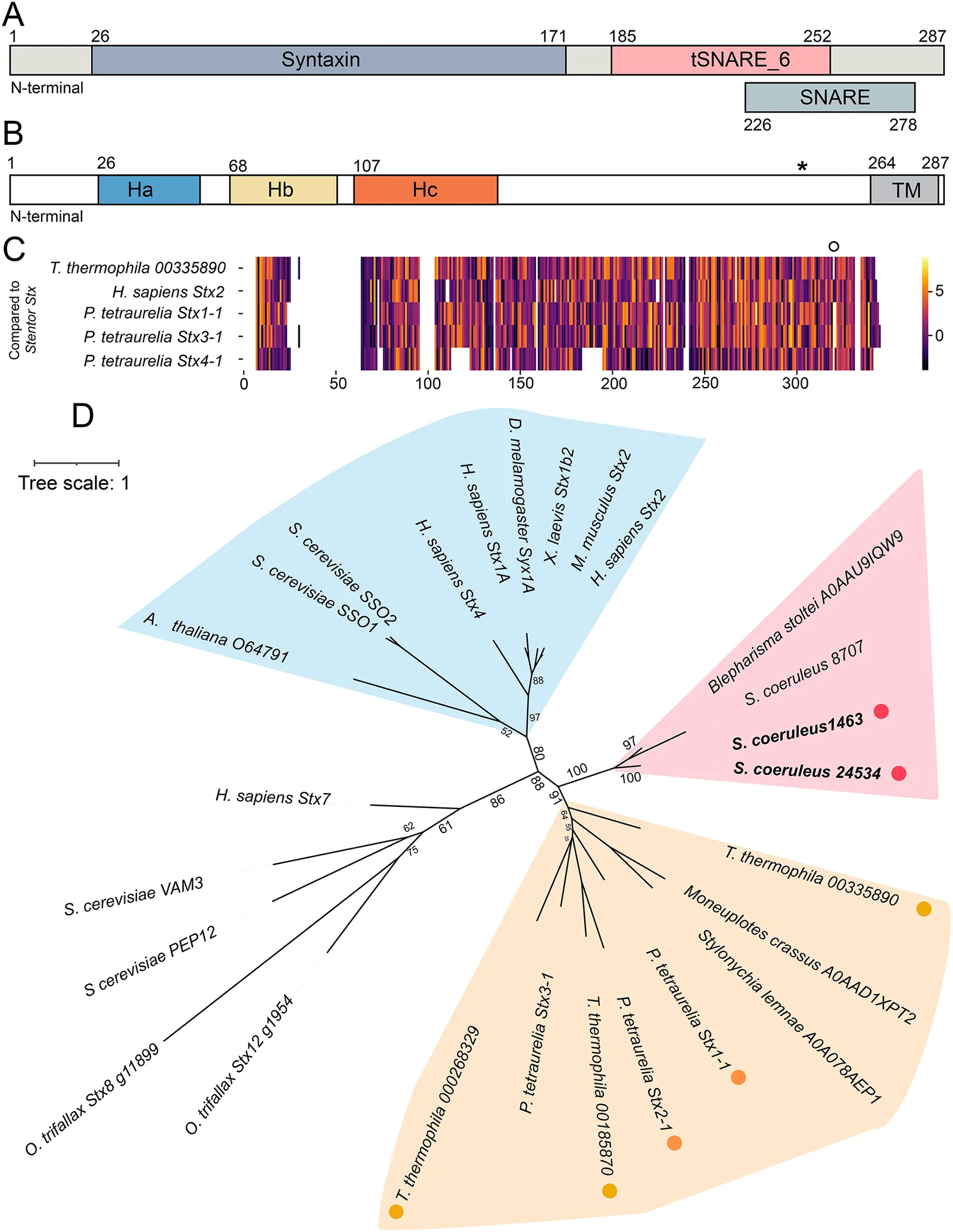
Domain structure and phylogeny of *Stentor* syntaxin. (A) InterPro domain analysis of the Stx protein revealed the presence of the syntaxin domain (slate blue), the tSNARE_6 domain (pink), and a conserved SNARE domain (light blue). Numbers indicate the amino acid residues of the protein. (B) Estimated positions of the regulatory helices Habc and the transmembrane (TM) domain in the *Stentor* Stx protein by multiple sequence alignment (MSA). The conserved Q residue is also indicated (*). (C) Heat map of the BLOSUM62 similarity score of the MSA of closely homologous syntaxin proteins from organisms (indicated), showing the conserved cysteine residues (yellow bars, hollow dot) at the C-terminus. (D) An unrooted phylogenetic tree of syntaxin proteins from organisms as indicated. Pink dots indicate *Stentor* Stx proteins. homologs in *Tetrahymena thermophila (yellow) and Paramecium tetraurelia (*orange dots) are indicated. The pink region shows clustering of syntaxins from the subphylum Postciliodesmatophora, class Heterotrichae. The yellow region encompasses ciliates from the subphylum Intramacronucleata. The blue region shows well-conserved relationships between exocytic human, fly, mouse, frog, and yeast syntaxins. Numbers at the nodes indicate confidence of observations (bootstrap values). Tree scale is a measure of evolutionary change per unit length.

Domain analysis of the Stecoe_24534 gene sequence revealed that the protein contained the syntaxin domain (PF00804), a t-(or target)-SNARE domain (SM00397), and a SNARE domain (PF05739; Figure 2A). The protein was 98.61% identical (283 out of 287 amino acid residues) to the homologous protein Stecoe_1463 by a BLAST search. The two genes shared 93% sequence identity with 5-10 stretches of contiguous 20-50 base pairs without a single mismatch. Therefore, it is reasonable to assume that our RNAi construct targeted both proteins. All uncovered features of the *S. coeruleus* Stx protein will also likely apply to this sister protein 1463.

Syntaxins are known to contain three helices (Ha, Hb, and Hc) at their N-terminal domains that are regulatory in nature (Fernandez *et al.*, 1998; Lerman *et al.*, 2000). We utilized previous structural work and multiple sequence alignment of syntaxin proteins to estimate the position of the 3 helices in the *Stentor* Stx protein (Figure 2B; Supplemental Figure S3A; Fasshauer *et al.*, 1998; Fernandez *et al.*, 1998). *Stentor* syntaxin also contains a highly conserved Q (glutamine) residue present in the ionic inner core of the synaptic fusion complex. Mutation of this residue impairs fusion complex function (Fasshauer *et al.*, 1998; Scales *et al.*, 2001). A multiple sequence alignment of closely related syntaxin proteins from *Stentor coeruleus, Tetrahymena thermophila* and *Paramecium tetraurelia* showed the presence of this residue in all proteins (Supplemental Figure S3, B and C).

We constructed a heat map of amino acid residue similarity across close homologs of *Stentor* Stx (identified in Supplemental Figure S3) using the BLOSUM similarity matrix (Figure 2C; Supplemental Figure S3B). A conserved pair of cysteine residues was present at the C-terminus of the Stx proteins (Henikoff and Henikoff, 1992). *Paramecium tetraurelia* Stx1-1 and Stx3-1, which have been shown to localize at the plasma membrane, also contained this dual cysteine motif, whereas *P. tetraurelia* Stx4, thought to function in transcytosis, contained one cysteine at this position (Kissmehl *et al.*, 2007). *Tetrahymena thermophila* syntaxin 00335890 also contained one cysteine at this position, with the other residue being methionine. This pair of cysteines is also present in the neuronal human Stx1A protein transmembrane domain (C271, C272) and functions as a putative palmitoylation site that could anchor the protein into the membrane or lipid rafts (Masaki *et al.*, 1998; Suga *et al.*, 2003; Kang *et al.*, 2008; Vardar *et al.*, 2022; An *et al.*, 2025). Due to the presence of a chain of aspartate residues in the human Stx1a that is absent in the *Stentor* Stx, the cysteines did not align with one another in a multiple sequence alignment (Supplemental Figure S3B). Mutation of these cysteine residues in Stx1a has been found to affect the kinetics of neurotransmitter release (Vardar *et al.*, 2022; An *et al.*, 2025). These residues could affect the conformation of the fusion complex or the kinetics of vesicle fusion (Vardar *et al.*, 2022; An *et al.*, 2025).

Next, we generated a phylogenetic tree to gain insight into the evolutionary relationship between *Stentor* Stx protein and syntaxin proteins of interest from other organisms. Because we only considered a limited number of syntaxin proteins (i.e., 26), our tree was “unrooted” and did not make conclusions about an evolutionary timeline or direction of descent, but only emphasized evolutionary relationships based on sequence similarity (Figure 2D). *Stentor* Stx protein (gene 24534) was most related to its sister protein (gene 1463).

*Stentor* falls within the class Heterotrichea of ciliates, and we found that *Stentor* Stx clusters with other heterotrich syntaxins (Figure 2D, pink region; Lynn, 2008; Gao *et al.*, 2016). Within the other major subphylum of ciliates (Intramacronucleata), we saw extensive interrelatedness of syntaxins (Figure 2D, yellow region), with syntaxins in the class Spirotrichea (genus *Stylonychia*, *Moneuplotes*) apparently emerging from a common ancestor. Clustering of proteins within a subphylum increased our confidence in the validity of our phylogenetic analysis. Relationships between other eukaryotes (human, mouse, fly, yeast) obeyed current phylogenetic models (Burki, 2014). Notably, syntaxins with defined roles in exocytosis or localization at the plasma membrane appeared related (Teng *et al.*, 2001), such as the yeast proteins SSO1&2 and human, mouse, and fly syntaxins 1,2 and human syntaxin 4 (Figure 2D, blue region). Putative endosomal/vacuolar syntaxins were found to cluster separately from the plasma membrane syntaxins, such as the human *Stx7* and the yeast (*S. cerevisiae*) VAM3 and PEP12 proteins (Wong *et al.*, 1998; Teng *et al.*, 2001).

### Syntaxin-deficient *Stentor* cells localize aberrant vacuoles at the cell surface

**FIGURE 3:**
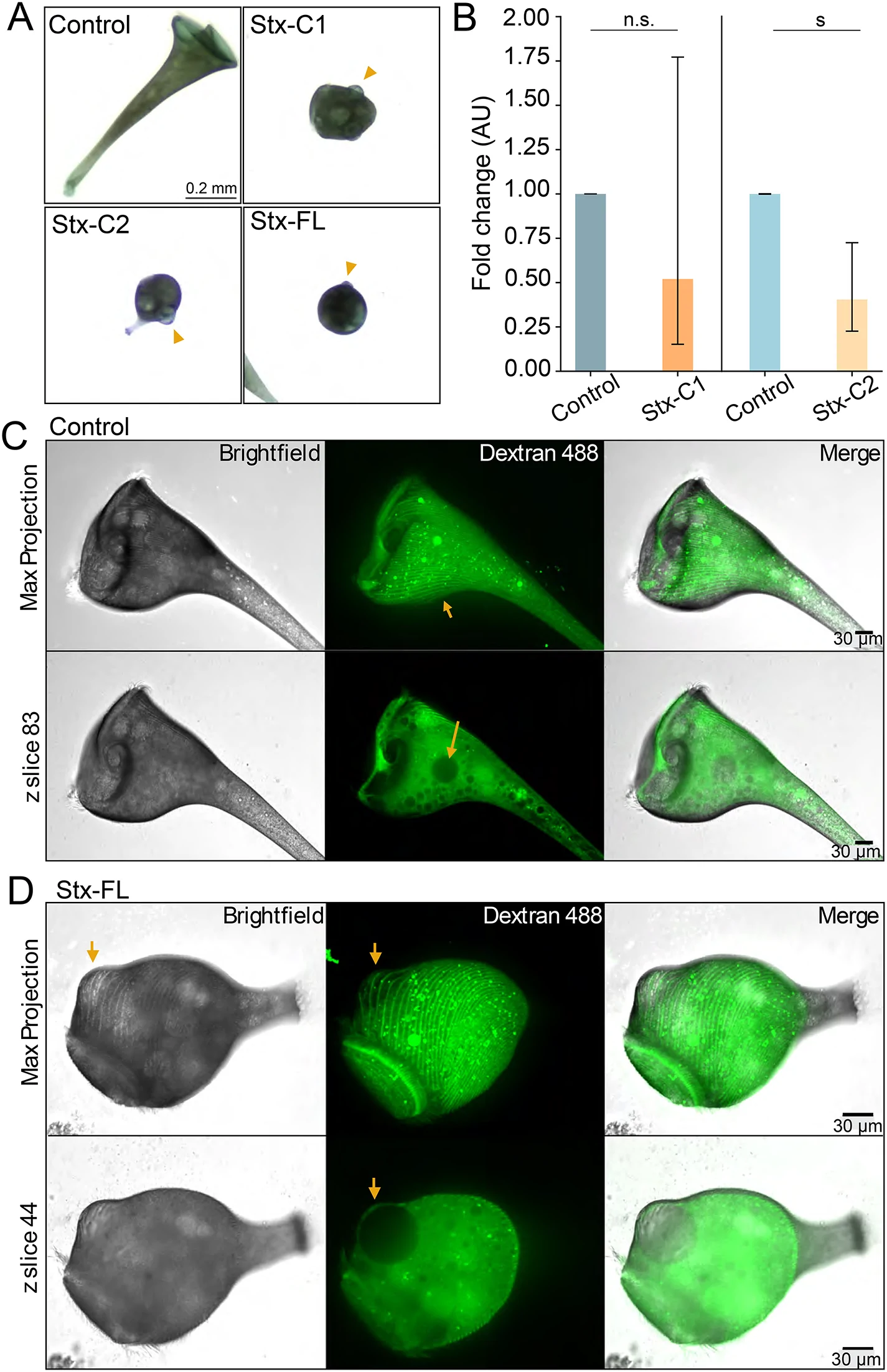
Syntaxin-deficient cells aberrantly localize vacuoles. (A) Brightfield images of control, Stx-FL, Stx-C1, and C2 (as indicated) depleted cells show aberrantly localized protrusions on the cell surface (yellow triangles) as a consistent phenotype between knockdown cells. (B) Fold change of gene expression in non-overlapping knockdown constructs is depicted. Average fold change for Stx-C1 was 0.52 with 95% C.I. of 0.152 to 1.772 (non-significant, n.s.), and average fold change for Stx-C2 was 0.404 with 95% C.I. of 0.226 to 0.725 (statistically significant, s., *n* = 3 replicates). (C) Representative images of syntaxin knockdown cells injected with Alexa-488 dextran reveal that the vacuoles are found relatively interior (z slice 83 - bottom panels) of control cells (arrows) and cortical rows are not perturbed by their presence (top panel). (D) In Stx-FL knockdown cells, vacuoles (arrows) are found proximal to the cell surface with cortical microtubules curving around them.

Having identified *Stentor* Stx as vital for post-wound cell survival, we next investigated protein function by perturbing the gene in native, unwounded cells. Syntaxin-deficient cells were characterized by the presence of aberrantly localized protrusions on the cell cortex, which we did not observe in control cells (Figure 3A). In addition to knocking down the full-length gene (which we will denote Stx-FL), we also carried out RNAi using non-overlapping RNAi constructs each covering different parts of the whole gene, which we denote Stx-C1 (residues 39-424) and Stx-C2 (residues 425-794). We observed the same aberrant vacuoles using RNAi with each of these non-overlapping constructs (Figure 3A; Supplemental Table S1). In contrast to control cells that had a characteristic conical shape, syntaxin-deficient cells were often smaller and rounded (Supplemental Figure S4). Upon manifesting the phenotype after seven days of RNAi, most cells showed conspicuously large vacuoles. Some cells were starting to die as judged by morphology and motility (Supplemental Figure S6C). However, many cells remained healthy, as judged by cell shape and motility, even though they showed the vacuole phenotype, and it was these healthy KD cells that were utilized in experiments. Until the vacuolation phenotype appeared at Day 7, cells underwent normal cycles of replication and growth with no differences from control cells (Supplemental Figure S6C). Due to the fact that our apparatus cuts a batch of cells into a reservoir from which cells are subsequently tested for survival, it has not been possible to determine whether the variation in cell volume or roundness documented in Supplemental Figure S4 correlates with the subsequent survival or death of individual cells.

We used qPCR to assess the extent of gene knockdown in cells targeted by the Stx-C1 and Stx-C2 constructs (see Methods). GAPDH transcripts were utilized for normalization (Slabodnick *et al.*, 2014) (Figure 3B). qPCR analysis revealed that syntaxin mRNA levels in cells transfected with the knockdown constructs (C1 and C2) were approximately 50% lower than in control cells (0.520x in cells targeted by the Stx-C1 construct and 0.404x for Stx-C2; Figure 3B; fold change and 95% C.I. reported).

To determine whether the protrusions on the cell cortex in syntaxin-deficient cells were blebs of the plasma membrane pulling away from the cytoskeleton or vacuoles deforming the surface, we microinjected Alexa-488-labeled dextran (m.w. 10,000 Da) in control and knocked-down cells. The dextran appeared to label the cortical microtubules (known as KM fibers; Huang and Pitelka, 1973) in some cells by an unknown mechanism. In control cells, vacuoles could be visualized as regions in the cytoplasm with low dextran fluorescence (Figure 3C). These vacuoles were found towards the cell interior relative to the cortical microtubules, and the microtubules were unperturbed by the vacuoles. In contrast, in Stx-FL knockdown cells, we observed large vacuoles that were pushed against the cell surface, with the KM fibers curving around them (Figure 3D). We confirmed this outward bulging of the KM fibers using immunofluorescence of control and knockdown cells (Supplemental  Figure S5). Our results indicate the presence of membrane-bound vacuoles close to the cell surface that are impermeable to dextran, and exclude the possibility that the structures are contiguous with the rest of the cytoplasm. The presence of unfused vacuoles at the cell surface in syntaxin-deficient cells is consistent with the protein's functions (Bennett *et al.*, 1992; Rothman, 1994; Teng *et al.*, 2001).

### Investigating the functional role of *Stentor* Stx in membrane patching

One possible function of syntaxin in wound recovery could be in membrane patching to close the wound in the plasma membrane. We previously used a membrane-impermeable dye exclusion assay to show that after wounding under the conditions used in these experiments, the half-life of membrane closure in *Stentor* cells is 100 s (Zhang *et al.* 2021), with cells dying if they failed to close their membranes within 1000 s. This indicates that rapid membrane healing is required for cells to survive more than a few minutes. We carried out a time-course of cell-survival post wounding in control versus Stx-FL RNAi cells, which revealed that all cells remained alive for at least 45 min post-bisection (Supplemental Figure S6, A and B). We observed the first events of cell death at 90 min, far longer than the inferred timescale for cell death if membrane patching had failed (Zhang *et al.* 2021). Qualitatively, cells became more vacuolated over time (Supplemental Figure S6A), dying gradually, with ∼75% of cells surviving at 6 h (consistent with our 12-h survival statistic in Figure 1). We also observed a similar vacuolation in a percentage of unwounded, syntaxin-deficient cells after the knockdown phenotype manifested (Supplemental Figure S6C). The fact that bisected cell fragments of syntaxin RNAi cells are capable of surviving for several hours argues against a gross or total defect in membrane patching.

Although our data suggest that there is not a wholescale loss of membrane patching in the stx-FL RNAi cells, additional experiments would be required to determine whether there may be subtle changes in the time required for wound closure compared with control cells. Our past experience with dye exclusion experiments found substantial variability between runs and a need for large numbers of cells, which is difficult in our knockdown experiments. Attempts to directly observe membrane patching dynamics by high-resolution imaging of cells confined in a PDMS microfluidic compression device (Zhang *et al.* 2024) failed due to the fact that Stx-FL cells burst during compression due to their large vacuoles. While we cannot rule out a possible delaying effect on membrane patching, our data argue against a major failure of membrane patching as the cause of death.

### Syntaxin cells show osmoregulatory defects in hypotonic conditions

**FIGURE 4:**
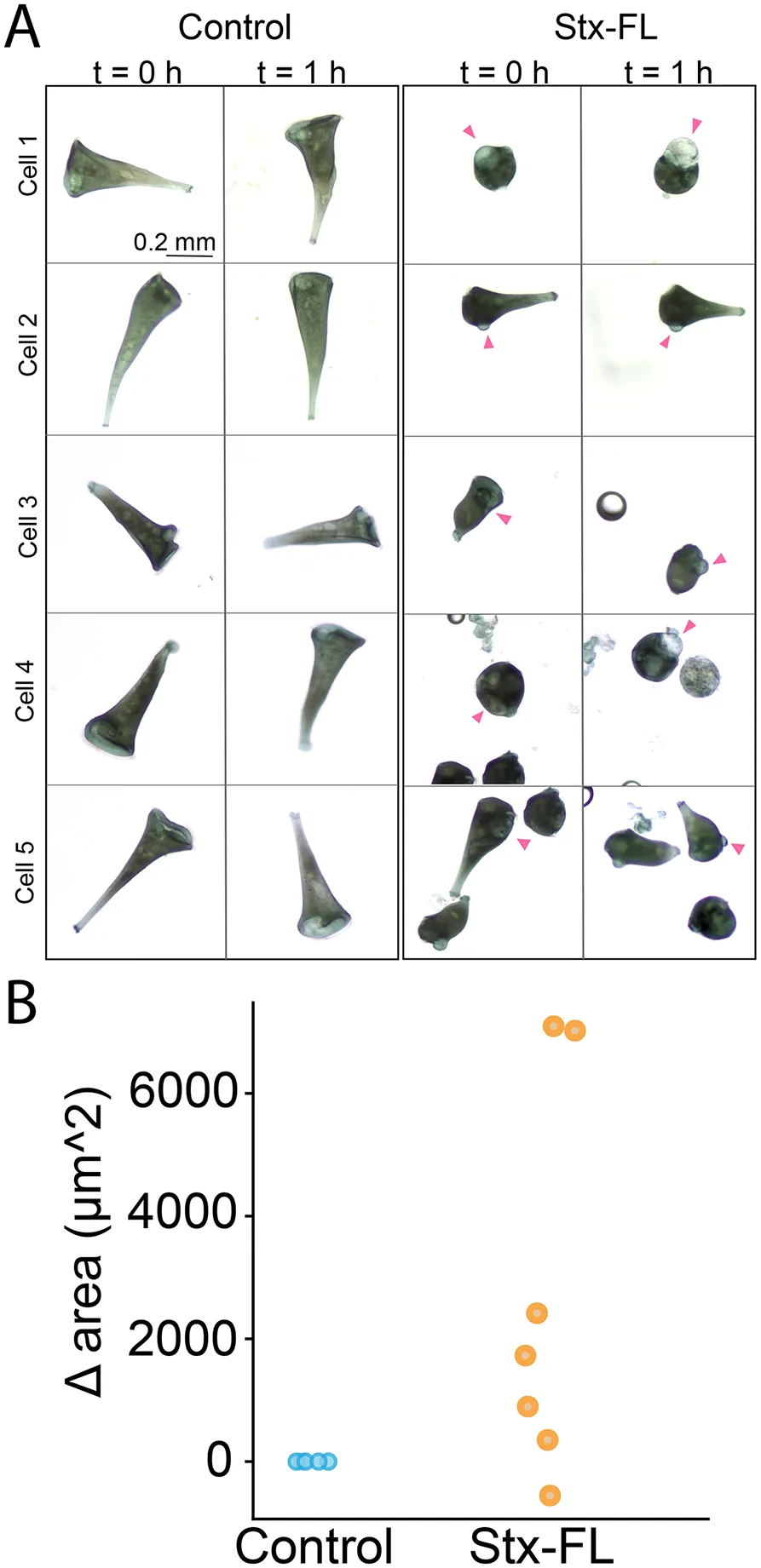
Syntaxin-deficient cells are not as osmotically adaptable as control cells. (A) Syntaxin-deficient cells (right panels as indicated) possess surface vacuoles (pink arrowheads) that often undergo swelling when cells are transferred from spring water (PSW) to de-ionized water having a lower osmolarity (0 h and 1 h time points depicted) as compared with control cells (left panels). Five separate examples are depicted. Scale bar is 200 microns. (B) Corresponding measured changes in projected area of the vacuoles in cells switched from PSW into de-ionized water. For cells lacking visible vacuoles (in control cells), the change in area was scored as zero.

Given that the main phenotype we could observe in Stx RNAi cells was the presence of large vacuoles (Figure 3, C and D), we hypothesized that deficiencies in vacuolar dynamics in Stx-FL RNAi cells could result in difficulties adapting to hypoosmotic stress. Ciliates, as with many freshwater-dwelling protists, rely on a contractile vacuole to expel water that enters the cell by osmosis. The contractile vacuole collects water from the cytoplasm and then expels it by transient exocytotic fusion of the vacuole at the plasma membrane (Cosgrove and Kessel, 1958; Allen, 2000; Velle *et al.*, 2023; More *et al.*, 2024). Membrane fusion is thus a key part of contractile vacuole function. Water accumulation is driven by proton pumps that drive water into the vacuole up its concentraiton gradient, while expulsion in many cases appears to be driven by hydrostatic pressure from the cytoplasm (Naitoh *et al.*, 1997; Velle *et al.*, 2023). Vesicular fusion and membrane dynamics are also involved in maintaining radial collection channels or tubules that feed into the central vacuole in some cell types (Allen, 2000; More *et al.*, 2024). The syntaxin family of proteins plays a role in contractile vacuole membrane dynamics in many organisms, including Paramecium (Kissmehl *et al.*, 2007; More *et al.*, 2024). To test for a role of syntaxin in osmotic regulation in *Stentor coeruleus*, we placed unwounded control and Stx-FL RNAi cells in hypotonic media (de-ionized water). While control cells were able to tolerate this osmotic challenge over 90 min, we observed accumulation of fluid into large vacuoles in Stx-FL RNAi cells (Figure 4A).

Measurement of vacuole size at *t* = 0 and 1 h showed that in most Stx-FL RNAi cells, vacuole size increased during exposure to hypotonic media (Figure 4B). Measurements of individual vacuoles before and after the hypotonic shift indicate that vacuoles in these cells start out with an average volume of 0.13 ± 0.08 nl SEM and then, after exposure to de-ionized water, they increase by an average of 0.37 ± 0.2 nl SEM. Given that the average volume of a Stentor cell is approximately 5 nl, we estimate that the vacuoles we see in Stx-FL cells account for roughly 3% of cell volume, and that the additional water accumulated in the vacuoles during the hypotonic shift, above what was already present in the Stx-FL vacuoles before the shift, is roughly 5% the volume of the whole cell.

The accumulation of plasma membrane-associated but unfused vesicles in Stx-FL RNAi cells, along with the growth of these vesicles when cells are transferred to hypotonic media, is consistent with the idea that these vacuoles may represent the contractile vacuole, which swells with water when the cell is placed in hypoosmotic conditions, but then cannot fuse with the plasma membrane to expel the contents.

If the vacuoles we see accumulating in Stx RNAi cells are indeed working as contractile vacuoles, this would suggest a plausible mechanism for the reduced viability post-wounding of Stx RNAi cells compared with control cells. The extracellular environment of freshwater protists is extremely hypotonic relative to the intracellular cytoplasm (More *et al.*, 2024). Cell injury may present an additional osmotic challenge if too much water enters the cytoplasm through the ruptured membrane, causing a fatal dilution of cell contents. Stentor cells must patch the membrane wound as well as excrete excess water and regulate the osmolarity of their cytoplasm.

Our attempts to directly infer or visualize water influx or volume changes post-bisection were unsuccessful, as high-speed flow of cells bisected by the stiff PDMS blade of the guillotine caused cells to lose cytoplasmic and membrane constituents at the blade site (Supplemental Figure S2 snapshots). Cut cells (both control and Stx-FL RNAi) lost close to half their volume during bisection (Supplemental Figure S4D). We could not therefore accurately estimate cell volume changes due to water influx in intact versus bisected cells.

We have not observed cell swelling or vacuole formation when wild-type cells are cut in our devices. To prominently visualize hypothesized water influx during wounding, we artificially increased the osmotic gradient during wounding. We pre-acclimatized cells in 75 mM sorbitol (refer to *Materials and Methods* section) and then bisected them into either the normal cell media, which is pasteurized spring water (PSW) or a 75 mM sorbitol solution (identical osmolarity to the pre-acclimation media). Sorbitol-acclimated cells bisected into PSW displayed prominent clear regions indicative of large vacuoles (Supplemental Figure S7A). These vacuoles persisted for up to three hours, after which their size decreased back down to baseline (Supplemental Figure S7B blue bars), and cells gradually returned to their normal shape. The transient increase in vacuole size is consistent with the possibility that they grew by accumulating excess water, while the loss of the vacuoles after several hours and the complete viability of cells after 24 h suggests that in cells with normal syntaxin function, the water can be expelled effectively. Similar vacuoles, forming and then disappearing on a similar timescale, also formed when sorbitol-acclimated cells were shifted directly into PSW without cutting (Supplemental Figure S7B black bars), but they were only about half the size of those that formed during cutting. Given that intact, uncut cells are on average twice the size of bisected cells, but form vacuoles that are half the size as those that form in the cut cells, we infer that cutting allows roughly four times the water to enter the cell and be collected in vacuoles compared with the amount that enters intact cells undergoing a similar osmotic shift.

### Wounding in an osmotically balanced medium increases survival of Stx-FL knockdown cells after cutting

Our results in Figure 4 indicated that the inability of Stx-FL RNAi cells to recover from wounding-associated osmotic changes could explain their low viability. If so, then it should be possible to reduce the lethal effects of the knockdown by reducing osmotic water entry into wounded cells. Osmotic stabilizers such as sorbitol are utilized in the isolation of delicate structures such as protoplasts to prevent water influx (Yang *et al.*, 2024). We asked if wounding the cells in a solution containing such osmotic stabilizers could help syntaxin-deficient cells to survive after wounding.

Ideally, in such experiments we would cut cells in media that is isosmotic with the cytoplasm, such that water would neither enter nor leave the cell during the time that the wound is open. The intracellular osmolarity of *Stentor* has not, to our knowledge, been directly reported. However, we expect it to be of the order of ∼100 mOsm/l based on previous measurements in other freshwater protists, which reported values of ∼85 mOsm/l for *Chlamydomonas* sp. (Milo *et al.*, 2010 Bionumbers Database BNID 112732), ∼90 mOsm/*L* for *Amoeba proteus* (Schmidt-Nielsen and Schrauger 1963), and of most direct relevance to *Stentor*, a range of 65–201 mOsm/l for the freshwater ciliate *Paramecium* (Stock *et al.*, 2001).

Unwounded control and Stx-FL RNAi cells tolerated the presence of 100 mM sorbitol in the cell media (PSW) for several hours. In these experiments, we noted shrinkage of the large vacuoles in syntaxin knockdown cells when placed into 100 mM sorbitol (Supplemental Figure S6E), indicating that 100 mM may be hyper-osmotic relative to the cytoplasm. We empirically determined a concentration of sorbitol (75 mM) that preserved cell viability overnight. After acclimatizing the cells in this medium for 30 min, we conducted a wounding survival assay in 75 mM sorbitol.

We observed that sorbitol-acclimated Stx-FL RNAi cells were injured but not bisected by the guillotine employed in our original survival assay comprising 90° blades (Figure 1), perhaps due to shrinking of the vacuoles of the Stx-FL RNAi cells in sorbitol or differences in cutting physics which are out of the scope of this work.

We therefore transitioned to a scaled-down guillotine where the channel was reduced by a factor of 2^1/3^ in each linear dimension to better match the reduced cell volume of Stx-FL sorbitol-treated cells, approximately half of the control cells (Supplemental Figure S4D). This scaled-down device incorporated angled blunt-to-sharp (bts) blades that have been empirically shown to bisect smaller cells efficiently (Koppaka *et al.*, 2021). Stx-FL RNAi PSW and sorbitol cells had comparable cell volumes, and we wanted to maintain consistent bisection conditions between the Stx RNAi cells. Therefore, we also used the 2^1/3^ bts guillotine to bisect Stx-FL RNAi cells in PSW. Normal-sized control cells (in PSW or sorbitol) were bisected using the standard-sized bts guillotine and showed 100% survival in all experiments. Importantly, the flow velocity at the blade was similar in all devices to ensure a consistent severity of wounding (refer to *Materials and Methods* section).

**FIGURE 5:**
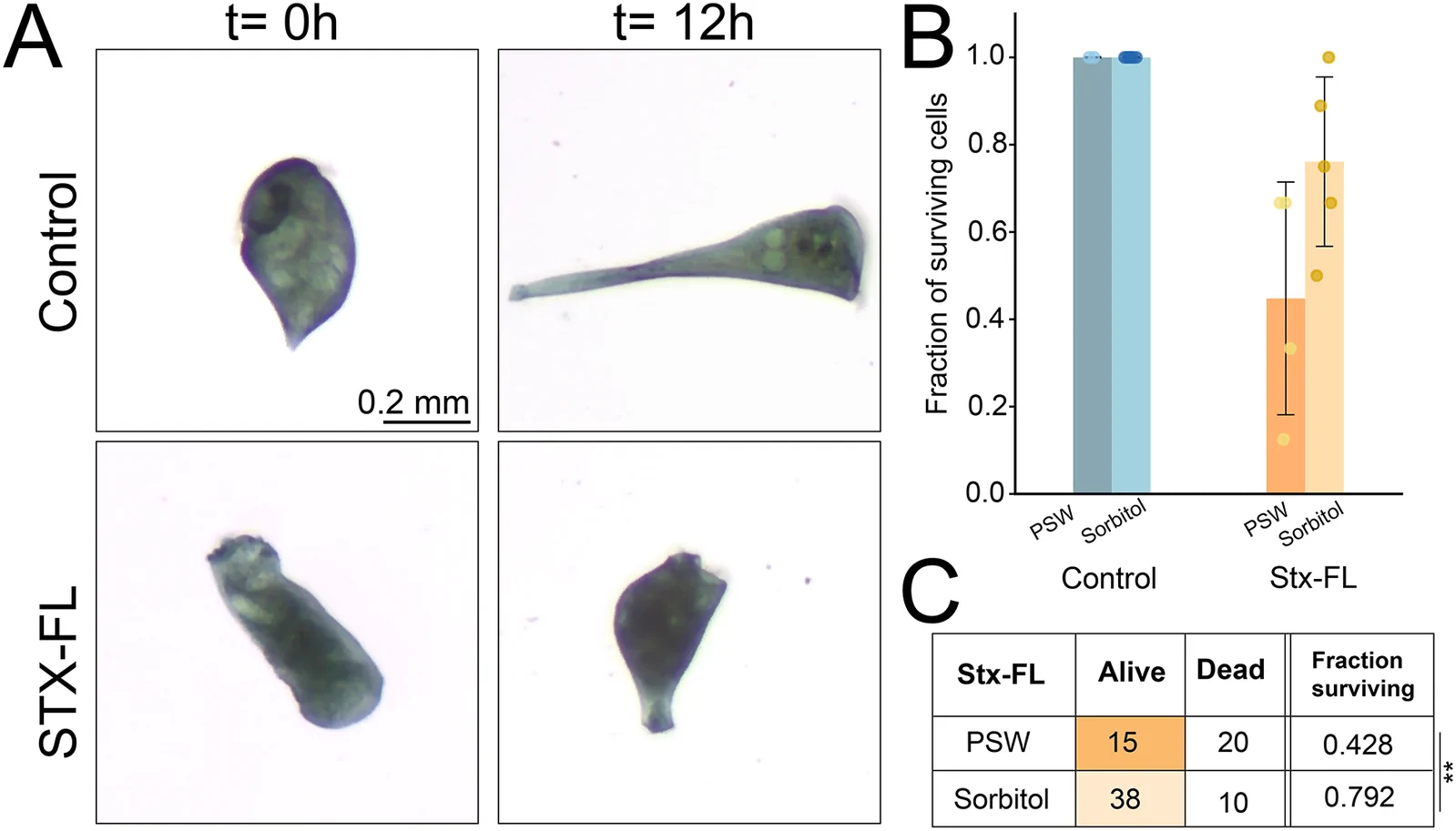
Bisection in osmotically-balanced conditions provides a mild survival advantage over wounding in spring water. (A) Control (upper) and Stx-FL RNAi (lower panels) were wounded in 75 mM sorbitol. Cells were visualized immediately after bisection (left panels) as well as 12 h post-wounding (right panels). (B) The fraction of surviving cells was quantified. Individual biological replicate experiments are denoted as filled circles. (C) The outcome of cell survival compared between Stx-FL RNAi cells in control (PSW- dark yellow) or isosmotic (sorbitol -light yellow) media, indicated by the total number of cell fragments across *n* = 4 biological replicates in each condition, is statistically significant by a two-sided Fisher's exact test (*p* = 0.001).

The assay was calibrated for the new blades and devices by measuring cell survival in normal cell media (PSW). Stx-FL cells once again fared worse than control cells, with the fraction of surviving cells being 0.448 for knockdown versus 1.0 for control cells. This was consistent with our observations in Figure 1 and our empirical evidence that bts blade provided a “gentler” bisection (Koppaka *et al.*, 2021). Acclimatization in 75 mM sorbitol provided a moderate survival boost for Stx-FL RNAi cells (fraction surviving increasing from 0.448 to 0.761 in PSW versus sorbitol), thus partially rescuing the post-wound survival of RNAi cells (Figure 5). A Fisher's exact test revealed that the difference in survival was statistically significant (*p* = 0.001; Figure 5C). We eliminated the possibility that the presence of sorbitol was affecting ciliary motility and thereby causing a false boost in survival (Supplemental Figure S8).

### Estimation of intracellular hydrostatic pressure

As the contractile vacuole accumulates water, it periodically expels the water by fusing with the plasma membrane to form a contractile vacuole pore, through which the water is squeezed out by hydrostatic pressure inside the cell (Naitoh et al 1997; Velle *et al.* 2023). The hydrostatic pressure is thus an important variable governing contractile vacuole activity. Given our hypothesis that syntaxin RNAi cells are unable to fuse with the plasma membrane to evacuate water, changes in vacuole size observed when these cells are placed in media with different osmotic activity provide a way to estimate the intracellular hydrostatic pressure, provided we make certain simplifying assumptions. We assume that the intracellular hydrostatic pressure P and the extracellular osmotic pressure both tend to drive water out of the cell, and the intracellular osmotic pressure drives water into the cell, such that at equilibrium




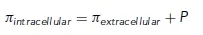




Assuming ideal dilute solutions and assuming that the osmotic coefficient Φ and the number of solute molecules n_i_ inside the cell are constant, the intracellular osmotic pressure is related to the osmotically exchangeable cellular water V_H2O_ as follows (Olmstead, 1966):




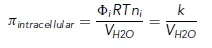




We have measured vacuole volume in three different media: de-ionized water, PSW, and 100 mM sorbitol. Denoting their osmotic pressures as 0, π_PSW_, and π_sorb_, respectively, we obtain the difference in the volume of osmotically exchangeable intracellular water that should be seen in each of two pairs of different conditions - PSW versus de-ionized water (Figure 4B), and PSW versus sorbitol (Supplemental Figure S6E):




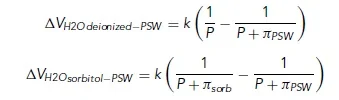




By taking the ratio, we can eliminate k.




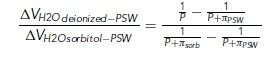




Based on our 2D images, we measured the cross-sectional area of the vacuoles under different conditions and converted those measurements to a volume assuming a spherical shape for the vacuoles. Given the measured volume change in the vacuole when cells are switched from PSW to de-ionized water (Figure 4B) and when cells are switched from PSW to 100 mM sorbitol (Supplemental Figure S6E), the ratio of the volume changes is approximately −13. We assume that k, which depends on the molecular composition of cytoplasm, is the same for both control cells (Supplemental Figure S6E) and syntaxin RNAi cells (Figure 4B). Solving for P gives a value of 0.9 mOsm/l, corresponding to roughly 2200 Pa, which is consistent with biophysical measurement of hydrostatic pressure in animal cells (Petrie *et al*., 2014; Perez-Gonzalez *et al*., 2019) as well as with the pressure range capable of driving water expulsion by the contractile vacuole (Velle *et al.*, 2023).

### Hypothesis for the role of syntaxin in Stentor wound healing

Cells deficient for the syntaxin gene died at significantly higher rates than control cells post-bisection. Syntaxin knockdown in unwounded cells led to aberrant vacuole localization, smaller cell size, and difficulty adapting to hypotonic solutions as compared with control cells. We do not know how to explain the smaller cell size but speculate that it might reflect effects on overall membrane trafficking during cell feeding or growth. We were able to partially rescue the post-wounding survival defect by wounding cells in a higher osmolarity solution.

Based on our data, we propose a speculative model in which wounding or injury breaches the barrier between the extremely hypotonic surroundings and cytoplasm, causing there to be a fatal dilution of the cell contents, even if the membrane is successfully repaired, unless the water can be expelled in a timely manner. We speculate that syntaxin-depleted cells die after wounding because they have defects in contractile vacuole-mediated osmoregulation (More *et al.*, 2024). In *P. tetraurelia*, syntaxin-2 (Syx2) has been found to localize to the contractile vacuole, and other syntaxins such as syntaxin-1 (Syx1) isoforms localize to the plasma membrane (Kissmehl *et al.*, 2007). *Stentor* syntaxin could either be directly involved in contractile vacuole function like Syx2 or play an upstream role in the osmoregulation pathway by mediating fusion of internal vacuoles that eventually merge with the contractile vacuole, after which the water is squeezed out, most likely by hydrostatic pressure (Velle *et al.*, 2023). Our estimate of hydrostatic pressure given above is consistent with such a function. Further details of the molecular process remain to be elucidated, and we therefore emphasize that our proposal that the syntaxin knockdown cells die after cutting due to an inability to remove excess water is at this point just a working hypothesis that will require further experimental work to test in detail.

Like single-celled freshwater ciliates such as *Stentor*, osmotic effects are also important during wound healing in multicellular organisms. For example, it has been shown that cell swelling due to influx of water and drop in interstitial osmolarity stimulates wound healing in zebrafish (Enyedi *et al.*, 2013; Gault *et al.*, 2014). Hypotonic stress causes phospholipase 2 to translocate to the nucleus, which in turn mediates the release of the inflammatory mediator arachidonic acid and recruits immune cells. In humans, extracellular osmolarity can vary during normal physiological processes as well as in pathological conditions such as hyperosmolar hyperglycemia and dilutional hyponatremia (Olmstead, 1966; Pasquel and Umpierrez, 2014). Our results suggest that such conditions might place additional stress on wound-repair pathways (potentially including syntaxins or other trafficking regulators) and compromise the ability of mechanically active cells such as skeletal or cardiac muscle cells to survive wounding. Although there exist fundamental differences in osmotic regulatory mechanisms in animals and *Stentor*, water transport may be a conserved aspect of wound recovery at the cellular level.

## MATERIALS AND METHODS

### Stentor cell culture

*Stentor coeruleus* were cultured in Pasteurized Spring Water (PSW; 132458, Carolina Biological Supplies) as previously described (Lin *et al.*, 2018; Zhang *et al.*, 2021). Cells were fed *Chlamydomonas reinhardtii* grown in a liquid suspension culture. A new feeding culture was inoculated every 7–10 d as described (Lin *et al.*, 2018).

### Cloning

The plasmid containing *Stentor* Stx (designated Stx-FL in the text) was generated by PCR (Radiant) using a sequence corresponding to nucleotide residues 34-794 of the coding sequence of the gene SteCoe_24534, a putative syntaxin protein. Non-overlapping RNAi constructs Stx-C1 and Stx-C2 corresponded to residues 39-424 and 425-794, respectively. Information about the gene sequences in the study is contained in Supplemental Table S1. Primer sequences are described in Supplementary Table S2. Genomic DNA was isolated from *Stentor coeruleus* by using either a phenol chloroform extraction protocol or the Quick-DNA/RNA Microprep Plus kit (Zymo Research - manufacturer's instructions). Briefly, for the phenol chloroform protocol, ∼200 cells were lysed into a buffer containing 100 mM Tris pH 9.0, 100 mM EDTA, and 1% SDS. After a 30 min incubation at 70°C, potassium acetate was added to a final concentration of 0.7 M. To the supernatant, one volume of phenol/chloroform/isoamyl solution was added (Sigma-Aldrich). DNA was precipitated using 0.7 volumes of isopropyl alcohol, washed with 70% ethanol, and resuspended in autoclaved water after drying. Genes were cloned from genomic DNA using Phusion Polymerase (New England Biosciences, manufacturer's instructions) and subcloned into the previously utilized pPR-T4P vector system using ligation-independent cloning (Aslanidis and de Jong, 1990; Adler and Alvarado, 2018). Plasmid products were sequenced by Elim Biopharma (Hayward, CA).

### RNA interference by feeding

RNA interference (RNAi) by feeding was carried out as described (Slabodnick *et al.*, 2014). Briefly, HT115 *E.coli* were transformed with plasmids containing genes of interest, grown, and induced with 1 mM IPTG. Bacteria were pelleted after 3 ½ hours, washed with PSW, and flash frozen in aliquots. An aliquot of frozen food equivalent to 200 µl of bacterial culture was fed per day to 100 starved *Stentor* cells (per condition). If using fewer cells, the food was scaled down proportionally. Phenotypes described were observed starting on day 6–7, and experiments were carried out on days 7–10. Cells were selected visually, using a dissection microscope, for experiments based on their small size, rounded morphology, and presence of unfused vesicles at the cell surface. A total of ∼15%–30% of cells yielded a visual phenotype corresponding to roughly 30–60 knocked-down cells per 200 *Stentor*. In the current study, candidate genes were knocked down using traditional RNAi by feeding. However, RNAi perturbation by microinjection has recently been shown to be a more effective method for genetic perturbation in *Stentor* (Kuecks *et al.*, 2025) and represents a future novel methodology for the study of syntaxin function in *Stentor*.

### Microfluidic devices

The 90° radial guillotine was fabricated in poly(dimethylsiloxane; PDMS) using standard photolithography as extensively described previously (Blauch *et al.*, 2017; Zhang *et al.*, 2021). The blunt-to-sharp guillotines were fabricated as described previously (Koppaka *et al.*, 2021). Briefly, channel mold designs were first created in SolidWorks (Dassault Systèmes), then fabricated 2-photon polymerization in IP-S photoresist on a silicon wafer. This process was performed using a 25X immersion objective (NA 1.4) in the Nanoscribe Photonic GT system (Nanoscribe, GmbH). Because of poor adhesion of the IP-S photoresist to the silicon substrate, only a single PDMS device was cast from the original mold. The cast PDMS device was then used to make a polyurethane replica. The polyurethane replica was used as the mold for subsequent devices. A smaller device scaled down by 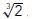 (with twice the number of parallel guillotines) was used for smaller syntaxin-deficient cells (only for experiments in Figure 5).

### Osmotic experiments

For the hypotonic experiments in Figure 4, cells were washed into and imaged in de-ionized water using the Zeiss Stemi 508 dissection microscope with the Axiocam 208 color camera for 0–90 min. For experiments in Figure 5 and Supplemental Figures S6 and S7, 100 mM and 75 mM concentrations of sorbitol were utilized (dissolved in PSW) where indicated. Vacuole cross sectional area (Figure 4B) were measured in Fiji. For calculating vacuole volumes, diameters and areas were measured in 2D images and then extrapolated to volumes assuming a spherical shape.

### Survival assay

Cells were collected and washed at least twice with PSW to reduce stickiness caused by RNAi food. For experiments in Figure 1 and Supplemental Figure S1, cells were injected into the radial microfluidic guillotine device using a fluidic pump at 1.4 cm/s per blade using a 10 cm-long outlet tubing and a fixed volume elution of 0.5 ml, as described previously (Zhang *et al.*, 2021). Each repeat survival assay provided us with a percent cell survival, so though we tested roughly 40–50 cell fragments on average, the total n ∼ 4 (indicated in each figure caption). As mentioned in the main text, live cells displayed ciliary beating of oral and body cilia, underwent helical “swimming” motions, or adhered to the bottom of plate wells. Cells were observed for as long as it was necessary to ascertain whether there was ciliary movement or the absence thereof. If the movement was difficult to discern, other authors or researchers viewed the cell to confirm the observations of the lead author. Due to the fact that our control cell survival was often 100% for every repeat, we couldn't verify normality of our data and therefore used the non-parametric Mann-Whitney test to measure statistical significance in Figure 1.

For the experiments shown in Figure 5, two media conditions were used, and microfluidic guillotines with angled blunt-to-sharp (bts) blades were used.

For the sorbitol experiments, control and Stx-FL RNAi cells were first acclimatized in 75 mM sorbitol in PSW for 30 min.

- Control cells were cut using a normal-sized 4-blade guillotine at 1.4 cm/s per blade.
- Stx-FL RNAi cells were cut using a guillotine that was scaled down in each linear dimension of the channel (height and width) by a factor of 2^1/3^, but that had twice the number of blades (8) with an average velocity of 1.1 cm/s per blade.

Flow velocity at the blade relative to the cell diameter was similar between the two devices, ensuring a consistent severity of wounding. Once eluted from the microfluidic guillotines, viable cell fragments were immediately counted at *t* = 0 h using a dissection microscope (Zeiss Stemi 508) equipped with an Axiocam 208 color camera. Cells were counted again at *t* = 12 h, with cellular or ciliary movement serving as indicators of viability.

### Microinjection

Microinjection was carried out as previously described (Kuecks *et al.*, 2025). Needles were pulled using a P-97 Flaming/Brown Micropipette Puller (Sutter Instruments). Needles were cut using a razor blade at an empirically determined distance to enable injection. 10 µl of injectant, 0.5 µg/µl or ∼ 50 µM fluorescently labeled Dextran Alexafluor 488 (Thermofisher, Catalogue number D22910) in PSW was spun down in a 1.5 ml Eppendorf tube for approximately 1 min, using a mini centrifuge, to remove clumps that may clog the needle tip. Needles were loaded with 0.5–1 µl of injectant approximately 5 min before injection. Approximately 50 (per condition) healthy control and knocked-down *Stentor* cells were starved for 12 h and placed in 1.5% methylcellulose solution to allow cells to shed their pellicle and enable easier injection. Microinjection was carried out using a Drummond Nanoject II system (discontinued) and monitored using a Zeiss Stemi 508 stereo microscope with a Transillumination 300 and dark field system.

### Live cell imaging

For images in Figure 1C and Figure 3A, cells were imaged in PSW using a Zeiss stereo microscope (Zeiss Stemi 508 with an Axiocam 208 color camera) in a 12 well-plastic or 24-well glass-bottomed plate. Microinjected cells were imaged using an inverted Nikon Ti2-E microscope equipped with a CREST X-Light V2 large field of view spinning disk, a Prime 95 25MM sCMOS camera, and a Celesta Light system. Figures 3C and D were imaged on this system using a Nikon CFI Plan Apochromat Lambda S 25XC Sil objective. Live cells were mounted in methylcellulose 1.5% solution and imaged in a 9 mm diameter, 120 µm thick spacer (Grace Bio Labs SecureSeal) between a standard glass slide and number 1.5 coverslip. Images were segmented, cropped, and registered using the OttoReg segmentation pipeline. OttoReg software was written using MATLAB (Mathworks).

### Bioinformatics

Domain analysis of the protein sequence Stecoe_24534 was performed using InterPro (Blum *et al.*, 2025). Domains were depicted using the Illustrator for Biological sequences and adapted using Adobe Illustrator (Xie *et al.*, 2022). Multiple sequence alignments were obtained using the protein sequences of the indicated genes and Clustal Omega or MUSCLE for alignments (Edgar, 2004; Sievers *et al.*, 2011; Sievers and Higgins, 2018). Sequence alignment in Supplemental Figure S3, A and B was generated using Jalview (Waterhouse *et al.*, 2009). Multiple sequence alignment depicted in Supplemental Figure S3C was generated using ggmsa (Zhou *et al.*, 2022). Biopython was utilized in the generation of the heatmap (Cock *et al.*, 2009). The phylogenetic tree was generated using IQ-TREE3, using the best-fit model and a bootstrap value of 5000 (Kalyaanamoorthy *et al.*, 2017; Hoang *et al.*, 2018; Wong *et al.*, 2026). Trees were visualized using FigTree (http://tree.bio.ed.ac.uk/software/figtree/) and iTOL (Letunic and Bork, 2007, 2024). Some fragments of code were generated with the assistance of ChatGPT, accompanied by extensive testing and debugging (https://chat.openai.com/). Fasta files, multiple sequence alignment files, and code will be made available upon request.

### RNA extraction and quantitative PCR (qPCR)

RNA was extracted from ∼50 *Stentor* cells (per condition) using the Quickprep DNA-RNA mini kit (Zymo) using manufacturer's instructions. On-column DNAse treatment was carried out at 1.5× the recommended volume (60 µl) and for 40 min instead of 15 min. RNA concentration was assayed using a ThermoScientific Nanodrop 2000. Primers used for qPCR are described in Supplemental Table S2. RNA template was diluted to ensure roughly equal amounts per reaction. qPCR was carried out using a BioRad CFX-Connect thermocycler, using the iTaq Universal SYBR® Green One-Step Kit as previously described (Slabodnick *et al.*, 2014) with an annealing temperature of 54°C. Controls lacking reverse transcriptase enzyme and template and a melt-curve were run at the end to ensure consistency between reactions and to verify the quality of RNA and product. GAPDH was used as a reference gene (Slabodnick et al, 2014). A standard curve for each primer pair was generated with an empirically determined threshold fluorescence level. Three replicates were run. The standard curve was used to determine the Cq and DNA amounts in ng for each condition. We obtained an average DNA amount in ng for control and knockdown conditions for both target (syntaxin) and reference (GAPDH) genes with associated standard error. Fold change was calculated by normalizing to the amount of the reference gene in each condition as follows:




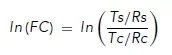




where T: target, R: reference, s: sample (knockdown), c: control.

Error was propagated using the formula:




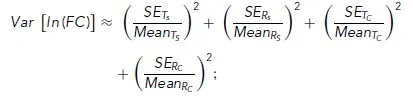




for independent samples. SE: Standard Error, Var: Variance. Confidence interval of *ln(FC)* = 1.96 ± SE.

### Cell shape properties and volume calculation

Aspect ratio and circularity were calculated from binarized light microscopy images of cells. All cells within a certain field of view were considered. Cell volume was computationally calculated using binarized high-magnification light microscopy images (Supplemental Figure S4D). First, the projected area silhouette of cells was centered at the centroid, and images were rotated in the direction of the lengthiest dimension of the cell. Integrated volume of each cell hemisphere on either side of the central line was calculated using coordinates at the cell's perimeter to compute the radius at each point (ra…rb, etc.).

### Data analysis, images, and figures

Data were graphed using Python packages numpy, matplotlib, and seaborn (Hunter, 2007; Harris *et al.*, 2020; Waskom, 2021). Other tools used were GraphPad online tools and Microsoft Excel. Statistical tests used are indicated in the figure legends. For Figure 1, control cell survival was often close to 100%, leading to a skewed distribution (by the Shapiro-Wilk test for normality), so a non-parametric Mann-Whitney U-test was used. For Figure 5, due to the fact that we were measuring only a ∼1.5× increase in Stx-FL RNAi survival from PSW into sorbitol and we had a small experimental n (*n* = 4), we pooled our cell survival data across biological replicates. This served to increase our statistical power as our experiments were unpaired, independent trials of cell survival with two possible outcomes (alive or dead). The dynamic range of images (using min-max) was adjusted using Fiji (Schindelin *et al.*, 2012). No quantification of data from modified images was carried out. Confocal images were scaled similarly to facilitate comparison. Non-similar modifications are indicated in the caption. Brightfield images were adjusted to enable visualization of cells against the background. Figures were made using Adobe Illustrator.

## Supporting information





## Abbreviations used:

BTS: blunt to sharp
CEP: centrosome protein
CI: Confidence interval
CV: contractile vacuole
ds: double stranded
Da: Daltons
EDTA: ethylenediaminetetraacetic acid
IPTG: Isopropyl β-D-1-thiogalactopyranoside
MFS: major facilitator superfamily
MSA: multiple sequence alignment
Pa: pascals
PDMS: polydimethylsiloxane
PSW: pasteurized spring water
qPCR: quantitative polymerase chain reaction
RNA: ribonucleic acid
RNAi: RNA interference
SDS: sodium dodecyl sulfate
SEM: standard error of the mean
Stx: syntaxin
Stx-FL: Syntaxin full length.
