# Supplementary material for: A role for the Stentor syntaxin protein in post-wound cell survival
Source: Mol Biol Cell. 2026 Jul 15;37(9):br29. doi: 10.1091/mbc.E25-11-0527 (PMC13525611; doi:10.1091/mbc.E25-11-0527)
Supplement: Supplementary file 1 [file mbc-37-br29-s001.pdf]

Supplemental Materials

Molecular Biology of the Cell

Nadkarni *et al*.

Supplementary information

Supplementary figures

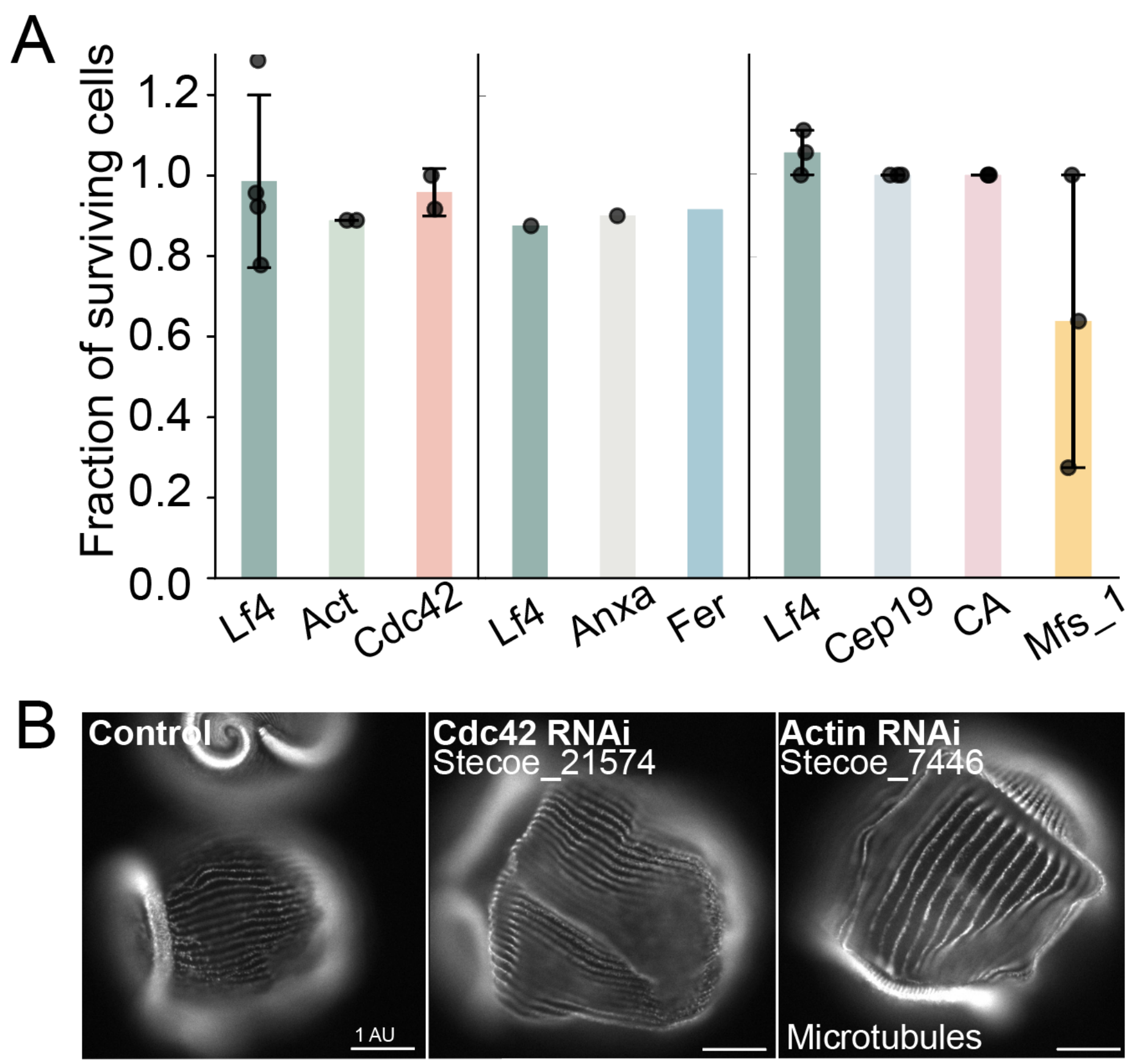

Figure S1. Survival assay of *S. coeruleus* perturbed for candidate wound-healing genes. (A) Fractional cell survival rates for the assay described in Figure 1. Genes indicated are as follows - Lf4 indicates a previously established control. Act - actin (Stecoe_7446), Cdc42 (Stecoe_21574), Anxa - annexin (Stecoe_18601). Fer - C2-domain containing protein- ferlin (Stecoe_31310), Cep19 - (Stecoe_12911), CA- carbonic anhydrase protein (Stecoe_37123), MFS_1- major facilitator superfamily 1 domain protein (Stecoe_168). Dots indicate individual experiments (biological replicates). Error bars represent S.D. (B) Representative confocal images of cells with indicated genes perturbed depicting no discernible gross morphological differences between control and knockdown cells.

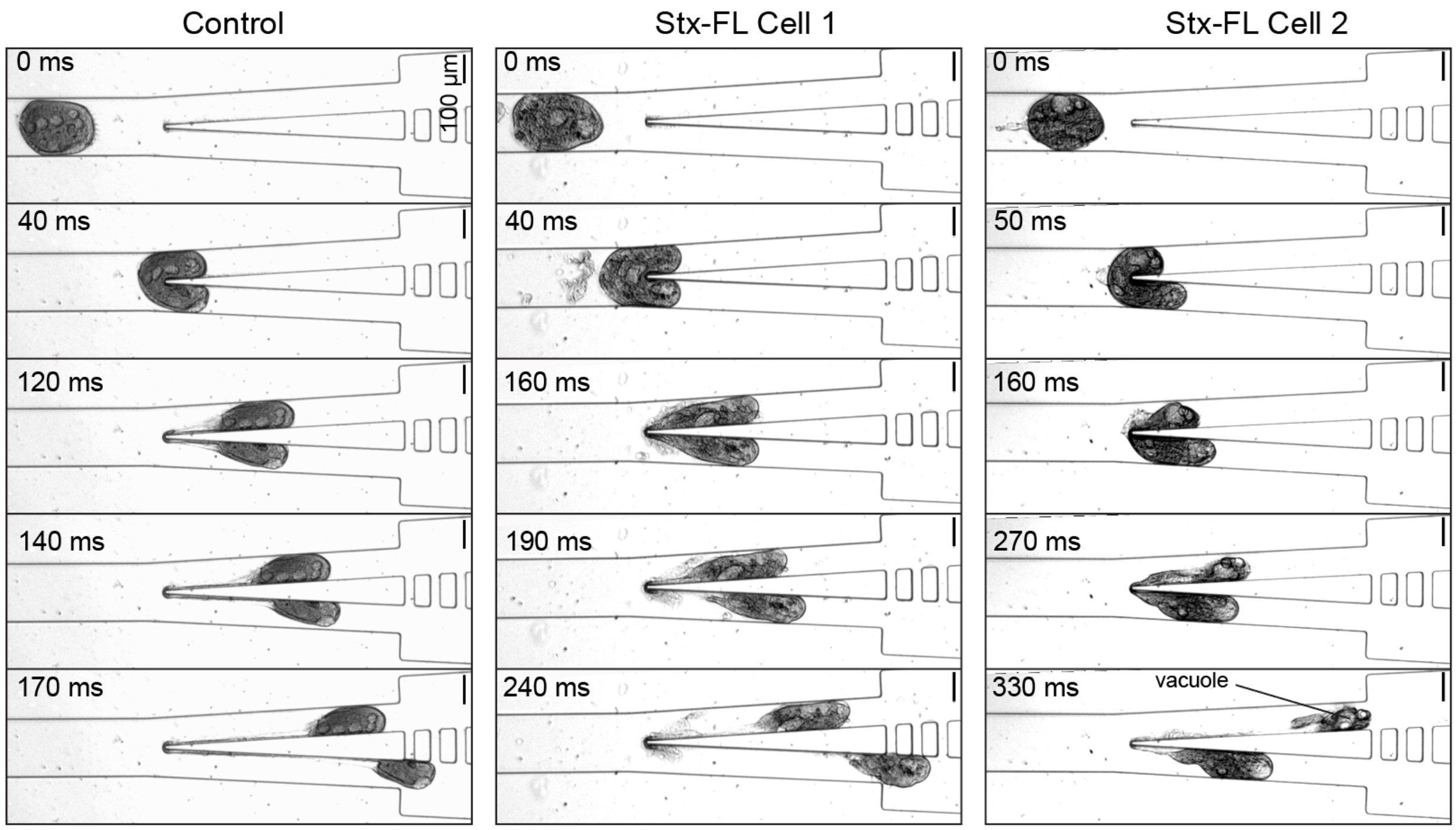

Figure S2. Montage of images of control (left panels) and syntaxin knockdown (Stx-FL) (middle and right panels) cells being bisected through the microfluidic guillotine. Scale bar is 100 µm.

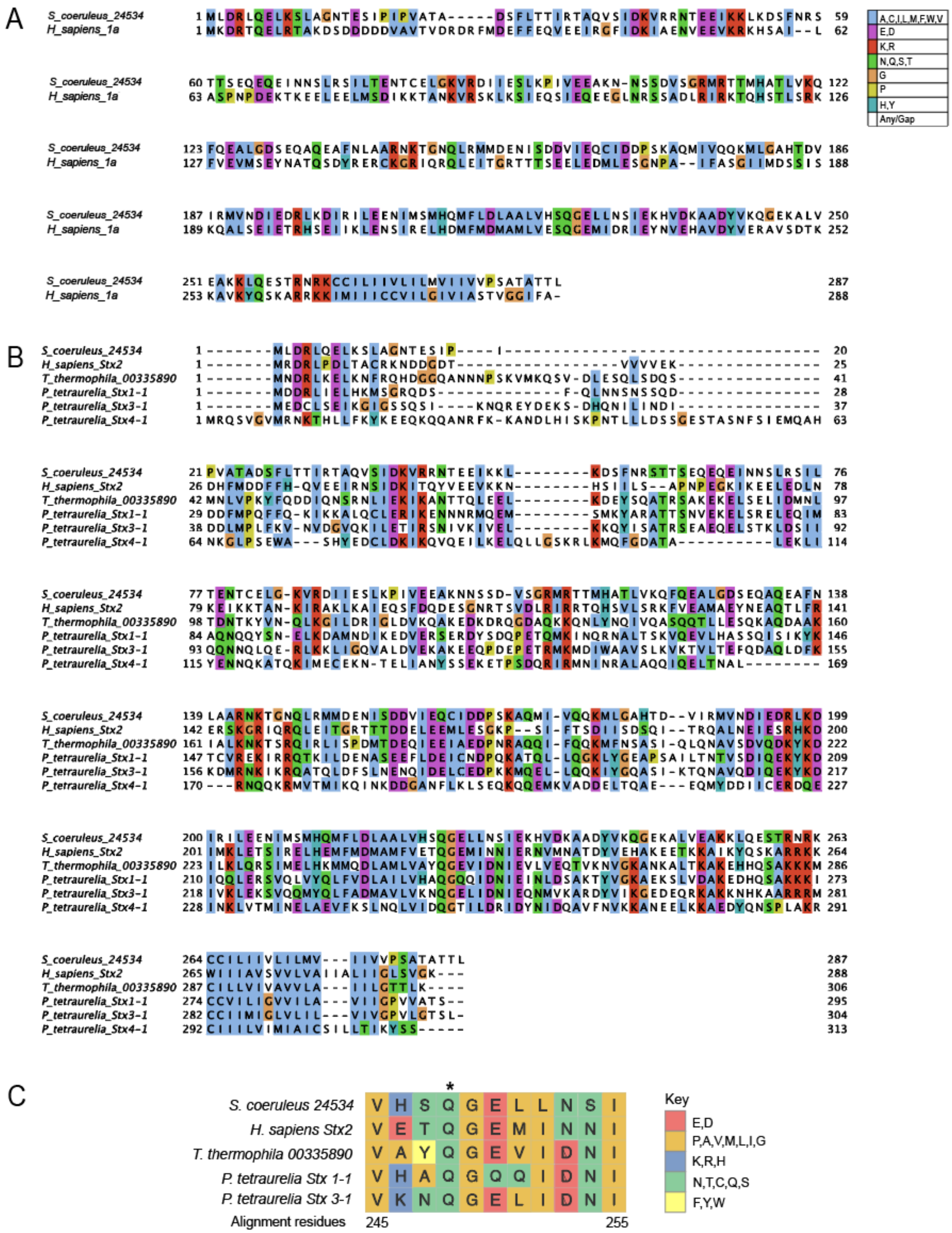
A
S_coeruleus_24534
H_sapiens_1a
A,C,I,L,M,F,W,V
E,D
K,R
N,Q,S,T
G
P
H,Y
Any/Gap
B
S_coeruleus_24534
H_sapiens_Stx2
T_thermophila_00335890
P_tetraurelia_Stx1-1
P_tetraurelia_Stx3-1
P_tetraurelia_Stx4-1
C
S. coeruleus 24534
H. sapiens Stx2
T. thermophila 00335890
P. tetraurelia Stx 1-1
P. tetraurelia Stx 3-1
Alignment residues
245
255
Key
E,D
P,A,V,M,L,I,G
K,R,H
N,T,C,Q,S
F,Y,W

Figure S3. Multiple sequence alignment of syntaxin proteins. (A,B) Species and genes are indicated in the figure. Amino acids are colored using the Clustal scheme, key denoted. (C) Multiple sequence alignment of close homologs of *Stentor coeruleus* syntaxin protein 24534 in *Homo sapiens, Tetrahymena thermophila* and *Paramecium tetraurelia* identified by BLAST show the degree of amino acid conservation around the Q glutamine residue (*).

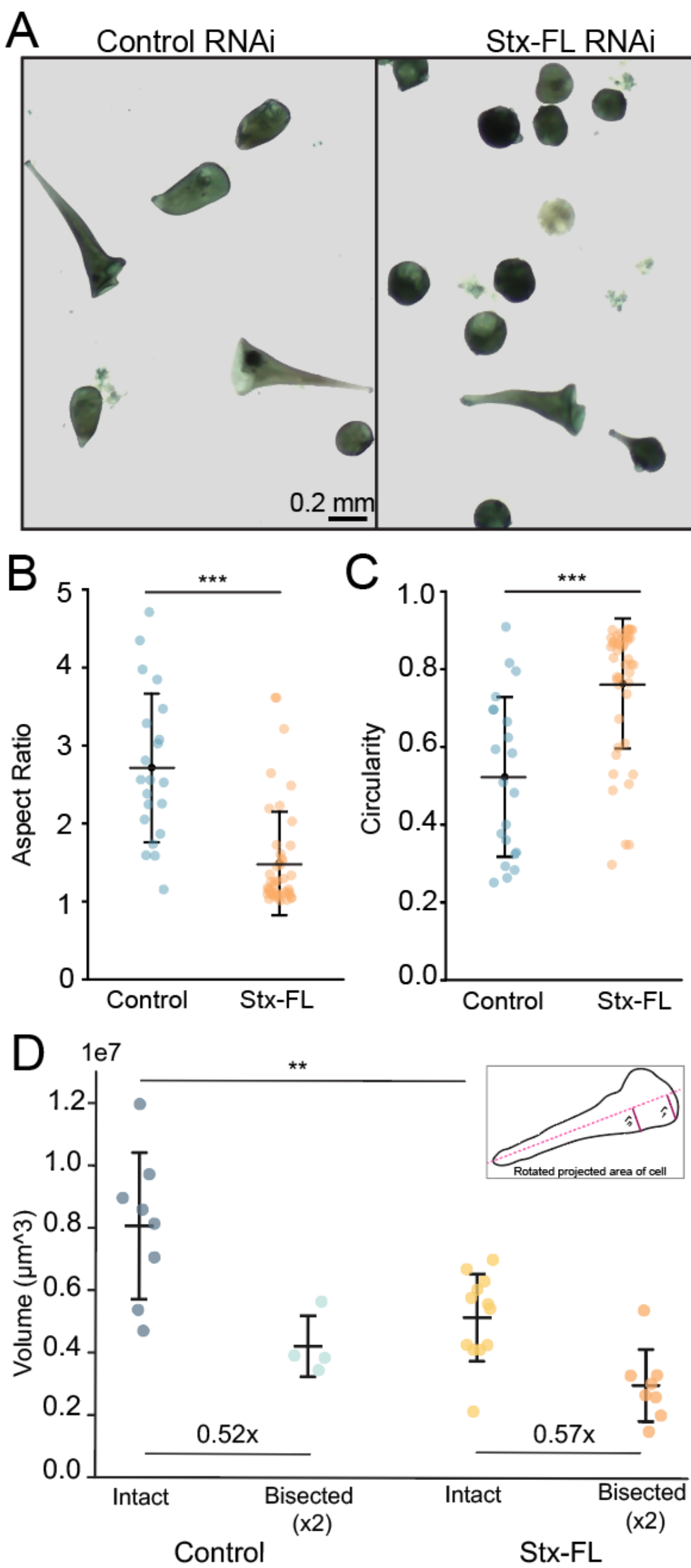

Figure S4. **Syntaxin depleted cells are on average more rounded and smaller than control cells.** (A) Brightfield images of unwounded control (left panel) and syntaxin deficient (right panel) cells and the resulting quantitative comparisons of shape parameters such as (B) aspect ratio (C) circularity of 2D projected areas of the cells showed that control cells had an aspect ratio of 2.718 ± 0.958 ($p<0.0001$) and an average circularity 0.522 ± 0.205 ($p<0.0001$),

versus knockdown cells, that had a much lower aspect ratio of 1.493 ± 0.671 and a circularity of 0.763 ± 0.167, indicating they were about half as elongated and ~1.5x more circular. (D) Computationally derived cell volume from projected cell area was derived by centering the 2D projected area silhouette a line passing through the centroid and in the direction of the lengthiest dimension of the cell (inset). Integrated volume of the cell hemisphere on each side of the central line was calculated using the coordinates of the cell periphery and the distance from the central line as radii (ra…rb, etc.). Cell volume for whole cells post bisection was also calculated (indicated on graph) and was found to be 0.52x (control) and 0.57x (Stx-FL RNAi) of unwounded cells prior to bisection on average, indicating a comparable amount of volume loss in both cases.

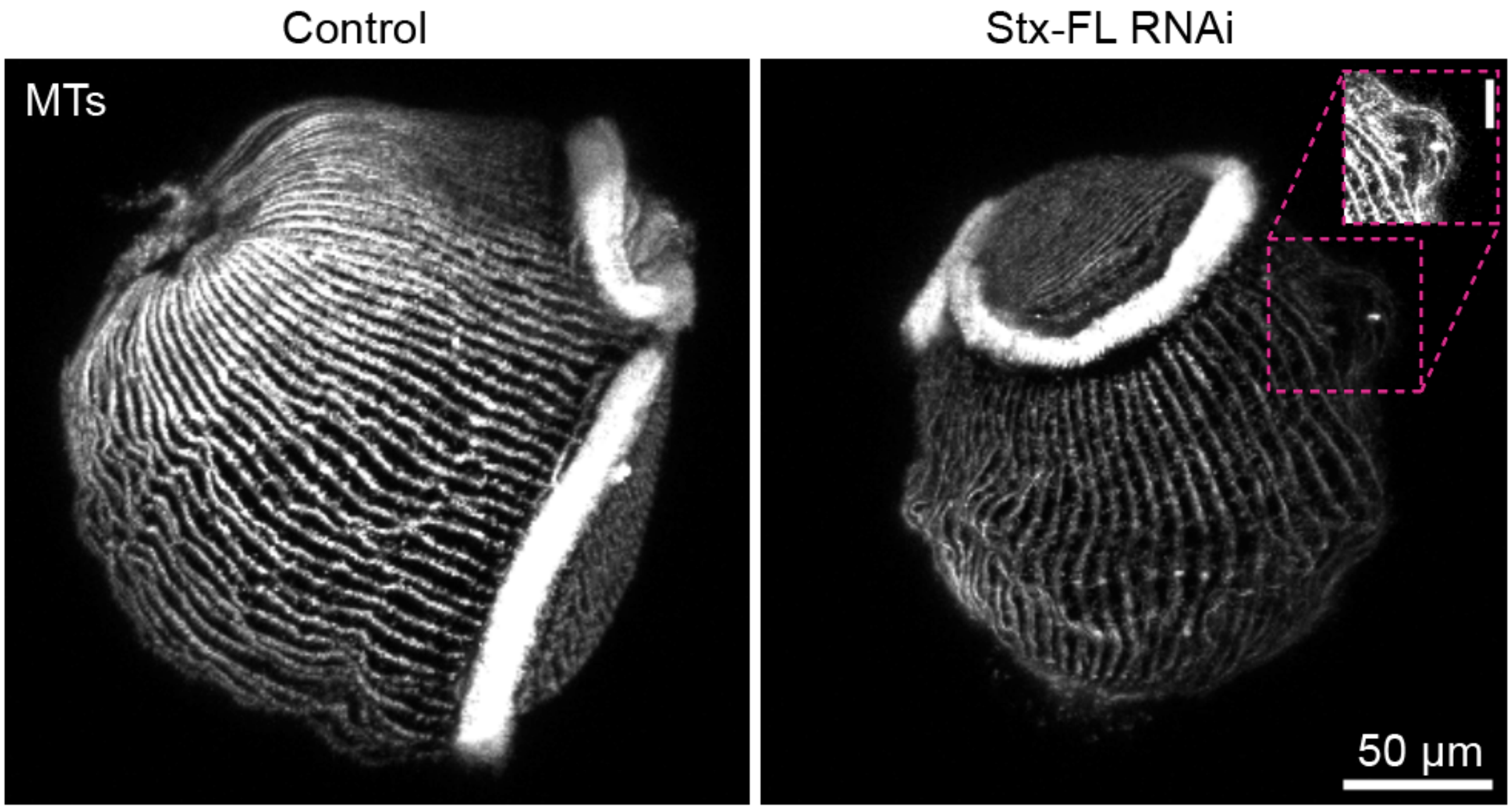

Figure S5. KM fibers curve around surface-level vacuoles in Stx-FL RNAi but not in control cells. Immunofluorescence of acetylated tubulin shows evenly spaced KM fibers with no aberrant curvature in control (left) panels and KM fibers curving around a vacuole (magenta box) in knockdown cells. Inset represents boxed area with enhanced dynamic range enabling visualization of the curved KM fibers. Scale bar in inset 10 µm. Representative images are from n=2 biological experiments.

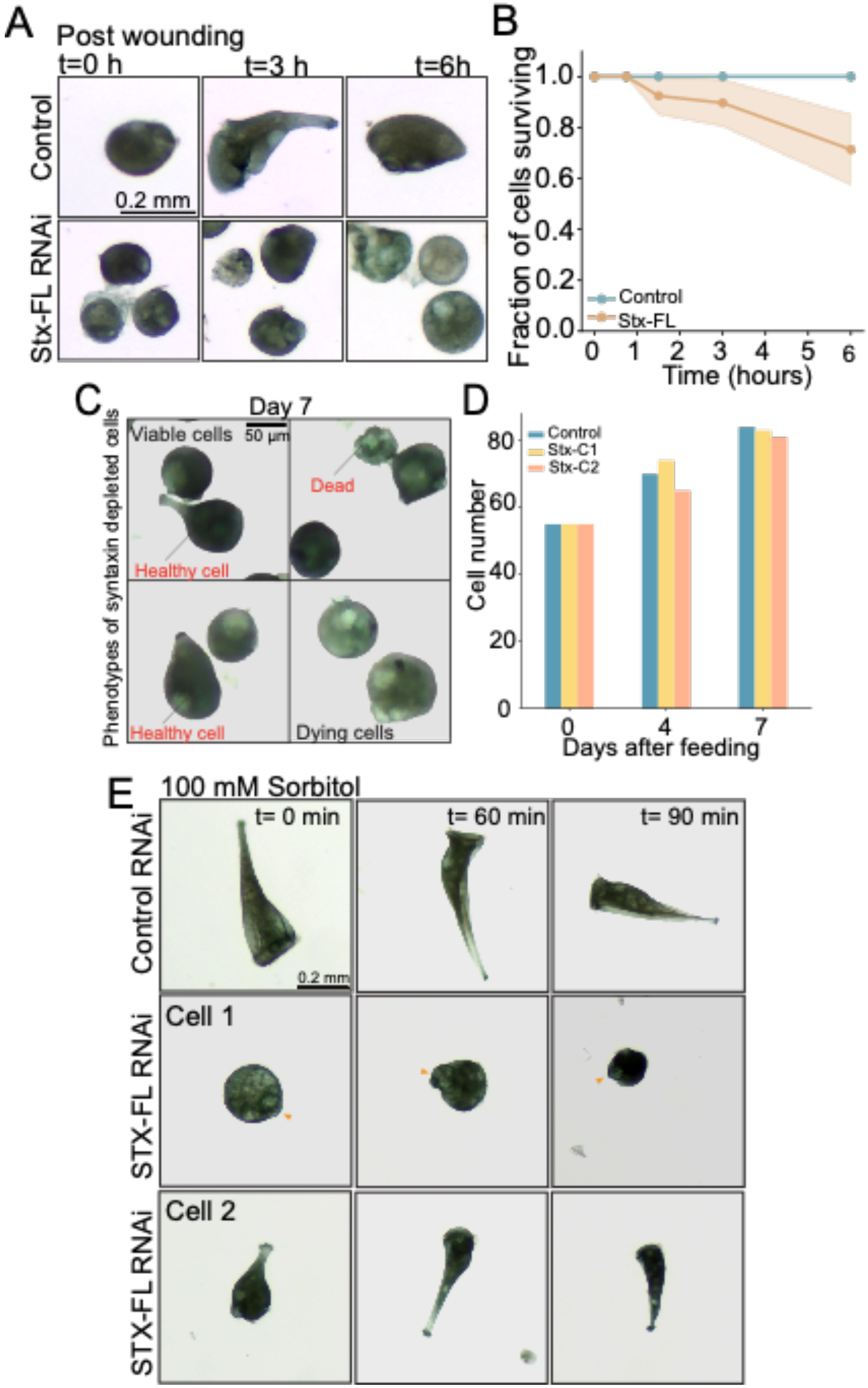
A
Post wounding
t=0 h
t=3 h
t=6h
Control
Stx-FL RNAi
0.2 mm
B
Fraction of cells surviving
1.0
0.8
0.6
0.4
0.2
0.0
Control
Stx-FL
0
1
2
3
4
5
6
Time (hours)
C
Day 7
Phenotypes of syntaxin depleted cells
Viable cells
50 µm
Dead
Healthy cell
Healthy cell
Dying cells
D
Control
Stx-C1
Stx-C2
Cell number
80
60
40
20
0
0
4
7
Days after feeding
E
100 mM Sorbitol
t= 0 min
t= 60 min
t= 90 min
Control RNAi
0.2 mm
Cell 1
STX-FL RNAi
Cell 2
STX-FL RNAi

Figure S6. Characterization of Stx-FL phenotypes. (A) Brightfield images of control and Stx-FL RNAi cells at various time points post wounding (indicated) show that Stx-FL RNAi but not control cells look increasingly vacuolated over time. (B) Graph depicting the timescale of survival of control (blue dots and lines) and Stx-FL RNAi (yellow) cells. Line represents mean and shaded region represents SD of n=3 biological replicates, average 10 fragments per experiment. (C) Vacuolation in unwounded Stx-FL cells after the phenotype expressed on Day 7. (D) Graph showing enumeration of cell division rates in control and knockdown constructs. (E) Brightfield images depicting control and Stx-FL RNAi cellular response to osmotically-balanced conditions (100 mM sorbitol) reveals shrinkage of the vacuole in cell 1 (yellow arrowheads) and a return to relative conical shape in cell 2.

A

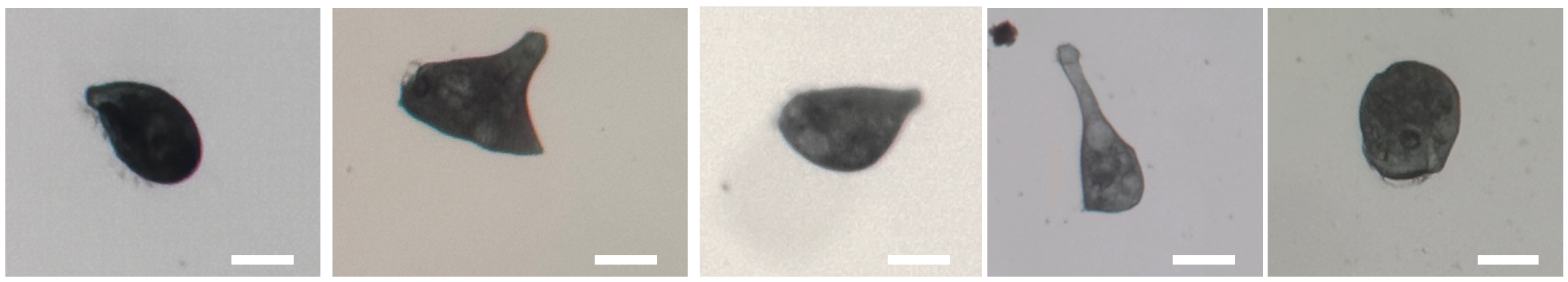

B

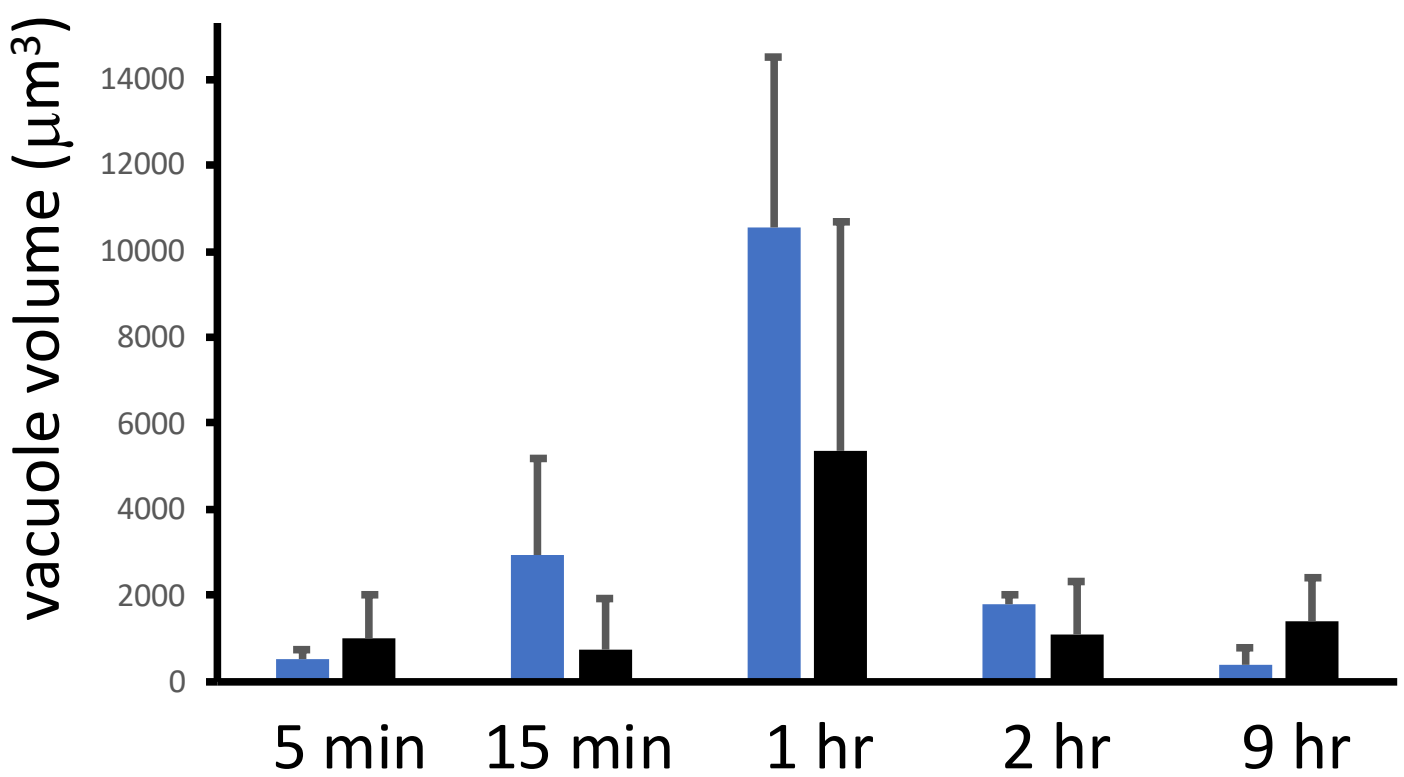

Figure S7. Forced entry of water into wt cells during cutting leads to transient vacuole accumulation. (A) Example images of wt control cells that were acclimated to 75 mM sorbitol and then cut into PSW. Scale bar 100 μm. (B) Average vacuole volume versus time after cutting into PSW (blue) or shift into PSW without cutting (black). Cells without visible vacuoles were scored as 0 volume. Error bars represent SEM.

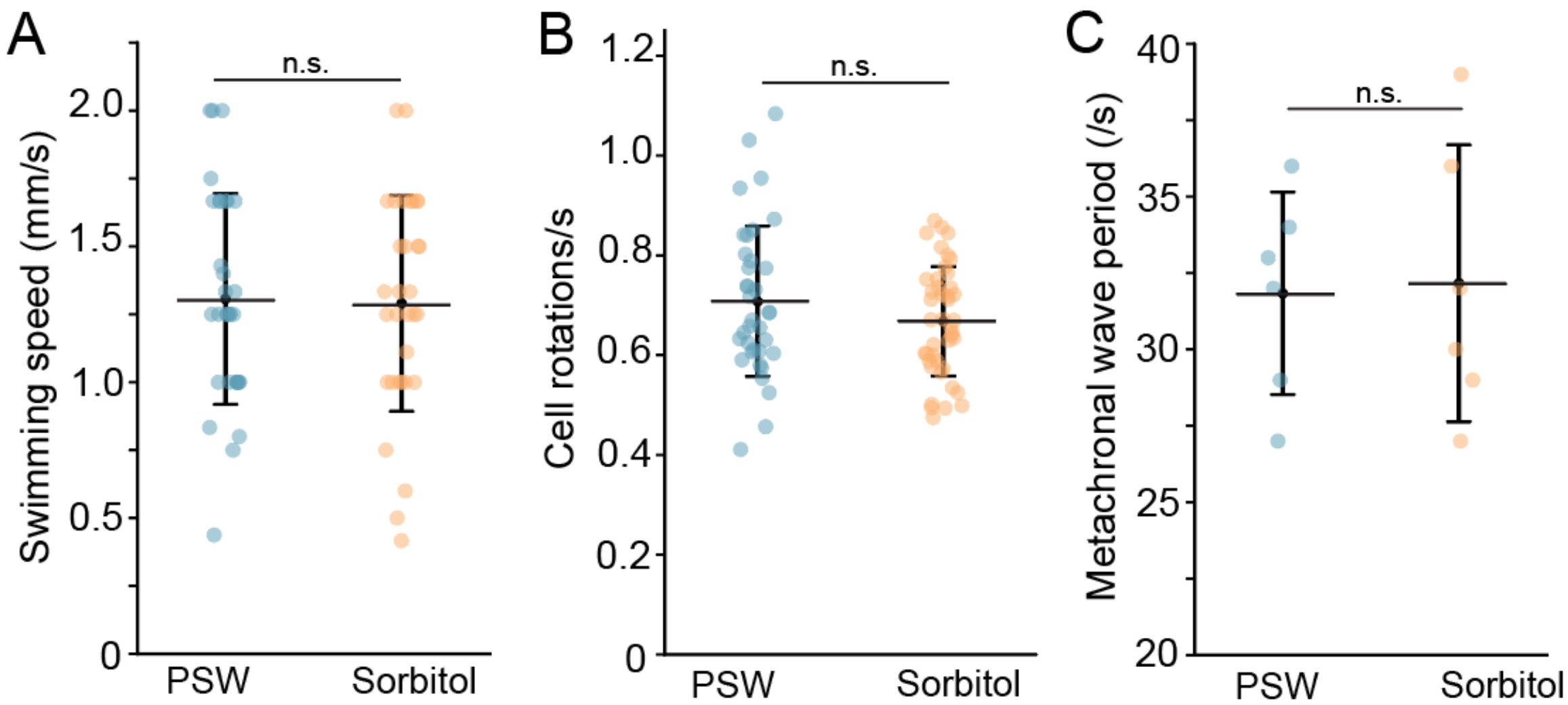

Figure S8. The presence of sorbitol in the cell medium does not affect ciliary motility. Graphs showing parameters of Stentor cells immersed in PSW alone or 75 mM in PSW (A) Quantification of swimming speed shows average speeds of 1.31±0.39 and 1.29±0.40 mm/sec respectively (n=32, p >0.05, n.s.) (B) Number of rotations per second showing averages of 0.71± 0.15 for control and 0.67 ± 0.11 rotations/sec respectively(n=36 and 43, n.s.). (C) Metachronal wave period for oral cilia of the membranellar band. Average period 31.9 ±3.0 and 32.2 ± 4.1 respectively (n.s.).

Supplementary table T1 Information about genes in the study

Gene/Protein	Functional significance/References	Stentor gene	Residues cloned (bases)	Primers Used (See table T2)
Stx_FL	Membrane fusion	Stecoe_24534	34-794	
Stx_C1	Membrane fusion	Stecoe_24534 First half	39-424	Stx_c1_f/r
Stx_C2	Membrane fusion	Stecoe_24534 Second half	425-794	stx_c2_f/r
Actin Domain Protein	Purse-string closure (Sonnemann and Bement 2011; Abreu-Blanco et al. 2011)	Stecoe_7446	12-1002	
Cdc42-like	Purse-string closure (Benink and Bement 2005)	Stecoe_21574	437-1100	
Annexin-like	Calcium-regulated protein, membrane transport, membrane repair in combination with dysferlin (McNeil et al. 2006; Gerke et al. 2024)	Stecoe_18601	26-239	18601_f/r
Ferlin (C2 domain protein)	Homologous to muscle dysferlin, acts in vesicle fusion, membrane repair in combination with annexins (Bansal et al. 2003; Lennon et al. 2003)	Stecoe_31310	1811-3408	31310_f/r
Cep19 domain protein		Stecoe_12911	4-265	12911_f/r
Carbonic anhydrase domain protein		Stecoe_37123	68-570	37123_f/r
Major Facilitator Protein_1		Stecoe_168	160-1170	168_f/r

Supplementary table T2 Primers utilized in the study

Primer Name	Purpose	Primer Sequence
Stx_c1_f	Subcloning	CATTACCATCCCGAATACAGAAAGCATC
stx_c1_r	Subcloning	CCAATTCTACCCGTTGCTGCAAGATTAA
stx_c2_f	Subcloning	CATTACCATCCCGAAATAAGACTGGGA
stx_c2_r	Subcloning	CCAATTCTACCCGATTTCCTATTCCTCGTC
**QPCR primers**		
Gapdh_f	ACGTGCTGGTGCTGCTAATA	
Gapdh_r	GACAGCGGCAACAACTTCAG	
C1_f	AGAAAGCATCCCAATCCC	
C1_r	GTTCTTGTTCACTGGTTGTT	
C2_f	ATGCTTGGAGCTCATACAG	
C2_r	GATAAAGCAGCGGATTATGTG	
**RNAi Screen**		
18601_f	Subcloning	CATTACCATCCCGCAGGACGACAGGGTAGATGG
18601_r	Subcloning	CCAATTCTACCCGAAGCTGCTTTCTAGCACGTAAA C
31310_f	Subcloning	CATTACCATCCCGTCAGATGCATCAGGTCTTGC
31310_r	Subcloning	CCAATTCTACCCGAACCCATCTTCAGGTTCACG
12911_f	Subcloning	CATTACCATCCCGTCGAAAGACTTCATGCCAAG
12911_r	Subcloning	CCAATTCTACCCGCCATTGATTCCCCAATAAGG
37123_f	Subcloning	CATTACCATCCCGAAGGTGGTGATGACTGGACTG
37123_r	Subcloning	CCAATTCTACCCGAAAGATGGGACCCGAAAGAG
168_f	Subcloning	CATTACCATCCCGCATTCAGGAAGGTTGGAAGG
168_r	Subcloning	CCAATTCTACCCGTCCGAAAAGAGCTCCAAATG
